# Updated list of the insect parasitoids (Insecta, Hymenoptera) associated with *Lobesia botrana* (Denis & Schiffermüller, 1775) (Lepidoptera, Tortricidae) in Italy. 2. Hymenoptera, Ichneumonidae, Anomaloninae and Campopleginae

**DOI:** 10.3897/zookeys.772.25288

**Published:** 2018-07-06

**Authors:** Pier Luigi Scaramozzino, Filippo Di Giovanni, Augusto Loni, Renato Ricciardi, Andrea Lucchi

**Affiliations:** 1 Department of Agriculture, Food and Environment, University of Pisa, via del Borghetto, 80 - 56124 Pisa, Italy

**Keywords:** Biological control, *Campoplex capitator*, European grapevine moth, ichneumonid wasps, natural enemies, taxonomy

## Abstract

In this second review of the parasitoids recorded on *Lobesia botrana* (EGVM) in Italy, an updated list and summary of the information available on 14 taxa of Ichneumonidae belonging to the subfamilies Anomaloninae and Campopleginae are provided. For each taxon, geographic distributions, host ranges, ecological role in viticulture and/or in other crops, and taxonomy are provided and discussed. For the most interesting species, tables summarizing the parasitization rates recorded in the field on EGVM or other lepidopteran pests are given. Identification mistakes and wrong synonymies that have generated great confusion and often made geographic distributions and host ranges unreliable are highlighted. A list of four Anomaloninae and 27 Campopleginae recorded on EGVM in Europe is also provided. Among the species examined, *Campoplex capitator* Aubert is the only potential candidate for biological control of EGVM.

## Introduction

A general overview of the parasitoids of *Lobesia botrana* (Denis & Schiffermüller, 1775) (European grapevine moth, EGVM) recorded in Italy, including Diptera Tachinidae and Hymenoptera Braconidae, has been recently published (Scaramozzino et al. 2017a). This second contribution deals with two subfamilies of Ichneumonidae, the Anomaloninae and Campopleginae.

## Materials and methods

As in the previous contribution (Scaramozzino et al. 2017a), the list of Ichneumonidae living on EGVM in Italy has been compiled using all the documents published on the subject, both in Italy (Table 1) and worldwide. As previously done, we also reviewed the parasitoid lists compiled by Thompson (1946), Coscollá (1997), Hoffmann and Michl (2003), and CABI (2017). The names of the species have been checked and updated according to the following databases: Taxapad (Yu et al. 2012), Noyes (2017) and Fauna Europaea (de Jong et al. 2014). In addition, material from the following private collections has been examined: J-F. Aubert at the Musée Cantonal de Zoologie in Lausanne (Switzerland), K. Horstmann at the Zoologische Staatssammlung in Munich (Germany, ZSM), F. Silvestri at the Department of Agriculture in Portici (Naples, Italy).

**Table 1.** Bibliography consulted for the compilation of the parasitoid list of EGVM in Italy. See paper references for the full bibliographic citation. The numbers on the left are the same as in Table 2.

1	Bagnoli B, Lucchi A (2006)
2	Boselli F (1928)
3	Catoni G (1910)
4	Catoni G (1914)
5	Catoni G (1915)
6	Colombera S, Alma A, Arzone A (2001)
7	Dalla Montà L, Marchesini E, Bagnoli B, Goggioli D (1993)
8	Del Guercio G (1899)
9	Delrio G, Luciano P, Prota R (1987)
10	Forti D (1991)
11	Leonardi G (1925)
12	Loni A, Samartsev KG, Scaramozzino PL, Belokobylskij SA, Lucchi A (2016)
13	Lozzia GC, Rigamonti IE (1991)
14	Lucchi A, Santini L (2011)
15	Luciano P, Delrio G, Prota R (1988)
16	Marchesini E (2007)
17	Marchesini E, Dalla Montà L (1992)
18	Marchesini E, Dalla Montà L (1994)
19	Marchesini E, Dalla Montà L (1998)
20	Marchesini E, Dalla Montà L, Sancassani GP (2006)
21	Nuzzaci G, Triggiani O (1982)
22	Pinna M, Gremo F, Scaramozzino PL (1989)
23	Roat C, Forti D (1994)
24	Ruschka F, Fulmek L (1915)
25	Scaramozzino PL, Loni A, Lucchi A, Gandini L (2017b)
26	Schwangart F (1913)
27	Schwangart F (1918)
28	Silvestri F (1912)
29	Stellwaag F (1921)
30	Stellwaag F (1928)
31	Zangheri S, Dalla Montà L, Duso C (1987)

## Results

From late 1800s to date, 120 ichneumonid species belonging to 51 distinct genera and ten subfamilies were reported on EGVM (Stellwaag 1928, Thompson 1946, Hoffmann and Michl 2003, Yu et al. 2012). In 1946, Thompson listed 60 ichneumonids associated to EGVM; since then, the number of species has almost doubled. Taxapad (Yu et al. 2012) reports 69 species, whereas Hoffmann and Michl (2003) provide the most complete list, with 94 species recorded. In our comprehensive list of 63 taxa reported for Italy (Table 2), the subfamily with the greatest number of taxa is Cryptinae (20), followed by Campopleginae (11), Pimplinae (10), Ichneumoninae (9), Metopiinae (7), Anomaloninae (3), Tryphoninae (2) and Cremastinae (1).

**Table 2.** List of Ichneumonidae (Hymenoptera) parasitoids of EGVM reported in Italy. Valid names are in bold. Synonyms, misspellings, combinations other than those valid today are preceded by a dot. Numbers are referred to Authors shown in Table 1. In two separate columns we indicated if the record is earlier or later than 1970. NORTH includes Aosta Valley, Piedmont, Liguria, Lombardy, Trentino-South Tyrol, Veneto, Friuli-Venezia Giulia and Emilia-Romagna regions; CENTER includes Tuscany, Marche, Umbria, Lazio and Abruzzo regions; SOUTH includes Campania, Molise, Apulia, Basilicata and Calabria regions.

Species	Subfamily	<1970	>1970	NORTH	CENTER	SOUTH	SICILY	SARDINIA
***Agrothereutes* sp.**	Cryptinae		[13] (*Spilocryptus* sp.), [22]	[13] (*Spilocryptus* sp.), [22]				
• *Agrothereutes abbreviator* (Fabricius, 1798) = emendation for *Agrothereutes abbreviatus* (Fabricius, 1794)								
***Agrothereutes abbreviatus*** (Fabricius, 1794)	Cryptinae		[16, 17, 19, 20, 23] (*A. abbreviator*)	[16, 18, 19, 20, 23] (*A. abbreviator*)				
***Agrothereutes pumilus*** (Kriechbaumer, 1899)	Cryptinae		[6, 9]	[6]				[9]
***Agrypon flaveolatum*** (Gravenhorst, 1807)	Anomaloninae	[26, 29, 30]		[26, 29, 30]				
• *Angitia tenuipes* Thomson, 1887 = syn. *Diadegma tenuipes* (Thomson, 1887)								
• *Angitia tibialis* (Gravenhorst, 1829) = syn. *Diadegma armillatum* (Gravenhorst, 1829)								
***Aptesis nigrocincta*** (Gravenhorst, 1815)	Cryptinae	[30] (*Helcostizus nigrocinctus*), [2, 26, 29] (*Microcryptus nigrocinctus*), [11] (*Microcryptus nigrolineatus*), [3, 4, 5, 28] (*Microcryptus nigrotinctus*)		[30] (*Helcostizus nigrocinctus*), [26, 29] (*Microcryptus nigrocinctus*), [3, 4, 5] (*Microcryptus nigrotinctus*)				
• *Atrometus geniculatus* (Holmgren, 1857) = syn. *Parania geniculata* (Holmgren, 1857)								
***Bathythrix argentata*** (Gravenhorst, 1829)	Cryptinae		[9, 16, 19, 20]	[16, 19, 20]				[9]
***Bathythrix decipiens*** (Gravenhorst, 1829)	Cryptinae		[16, 17, 18, 19, 20, 32]	[16, 17, 18, 19, 20, 32]				
• *Cambrus inferus* = misspelling for *Gambrus inferus* Thomson, 1896								
***Campoplex* sp.**	Campopleginae		[13, 22]	[13, 22]				
***Campoplex borealis*** (Zetterstedt, 1838)	Campopleginae		[21]			[21]		
* ***Campoplex capitator*** Aubert, 1960	Campopleginae	[3, 4, 5, 11, 28, 30] (*Omorgus difformis*), [2] (*Omurgus difformis*)	[1, 6, 7, 12, 14, 16, 17, 18, 19, 20, 25]	[6, 7, 16, 17, 18, 19, 20], [3, 4, 5, 28] (*Omorgus difformis*)	[1, 12, 14, 25]	[28, 30] (*Omorgus difformis*)		
* ***Campoplex difformis*** (Gmelin, 1790)	Campopleginae	[24, 26, 29, 30] (*Omorgus difformis*)	[21]	[24, 26, 29, 30] (*Omorgus difformis*)		[21]		
• *Cinxaelotus erythrogaster* Holmgren, 1890 =syn. *Dicaelotus erythrogaster* (Holmgren, 1890)								
***Cryptus* sp.**	Cryptinae	[13]		[13]				
***Diadegma armillatum*** (Gravenhorst, 1829)	Campopleginae	[28, 30] (*Angitia tibialis*)				[28, 30] (*Angitia tibialis*)		
***Diadegma tenuipes*** (Thomson, 1887)	Campopleginae	[2, 3, 4, 5, 11, 26, 28, 30, 31] (*Angitia tenuipes*)		[3, 4, 5, 26, 30, 31] (*Angitia tenuipes*)				
***Diadromus collaris*** (Gravenhorst, 1829)	Ichneumoninae	[30] (*Thyraeella collaris*), [28] (*Thyrella collaris*)				[30] (*Thyraeella collaris*), [28] (*Thyrella collaris*)		
***Dicaelotus* sp.**	Ichneumoninae		[13, 22, 23]	[13, 22, 23]				
***Dicaelotus erythrogaster*** (Holmgren, 1890)	Ichneumoninae	[2, 3, 4, 5, 11, 24, 26, 28, 29, 30, 31] (*Cinxaelotus erythrogaster*)		[3, 4, 5, 24, 26, 29, 30, 31] (*Cinxaelotus erythrogaster*)				
***Dicaelotus inflexus*** Thomson, 1891	Ichneumoninae	[2, 4, 5, 7, 28, 30, 31] (*D. resplendens*)	[1, 6, 16, 17, 18, 19, 20], [9, 32] (*D. resplendens*)	[6, 7, 16, 17, 18, 19, 20], [4, 5, 28, 30, 31, 32] (*D. resplendens*)	[1]	[28, 30] (*D. resplendens*)		[9] (*D. resplendens*)
***Dicaelotus pusillator*** (Gravenhorst, 1807)	Ichneumoninae	[8, 11, 28, 29, 30]		[29, 30]				
• *Dicaelotus resplendens* Holmgren, 1890 = misidentification for *Dicaelotus inflexus* Thomson, 1891 (see Marchesini and Dalla Montà 1994)								
***Endromopoda detrita*** (Holmgren, 1860)	Pimplinae	[2, 3, 4, 5, 11, 26, 28, 29, 30, 31] (*Pimpla detrita*)		[3, 4, 5, 26, 28, 29, 30, 31] (*Pimpla detrita*)				
***Exochus* sp.**	Metopiinae		[1]		[1]			
***Exochus lentipes*** Gravenhorst, 1829	Metopiinae	[29] (*E. notatus*)	[25] (*E. notatus*)	[29] (*E. notatus*)	[25] (*E. notatus*)			
• *Exochus notatus* Holmgren, 1858 = syn. *Exochus lentipes* Gravenhorst, 1829								
***Exochus tibialis*** Holmgren, 1858	Metopiinae	[2, 4, 5, 26, 29, 30, 31]	[13, 16, 18, 19, 20, 22]	[4, 5, 13, 16, 18, 19, 20, 22, 26, 29, 30, 31]				
• *Eulimneria alkae* (Ellinger & Sachtleben, 1928) = syn. *Sinophorus turionum* (Ratzeburg, 1844)								
• *Eulimneria crassifemur* (Thomson, 1887) = syn. *Sinophorus crassifemur* (Thomson, 1887)								
• *Eulimneria ramifemur* = misspelling for *Sinophorus crassifemur* (Thomson, 1887)								
• *Gambrus infernus* = misspelling for *Gambrus inferus* Thomson, 1896								
• *Gambrus inferus* Thomson, 1896 = syn. *Gambrus ornatus* (Gravenhorst, 1829)								
***Gambrus ornatus*** (Gravenhorst, 1829)	Cryptinae	[26] (*Cambrus inferus*), [29] (*G. infernus*), [2, 3, 4, 5, 11, 28, 30, 31] (*G. inferus*)		[26] (*Cambrus inferus*), [29] (*G. infernus*), [3, 4, 5, 30, 31] (*G. inferus*)				
***Gelis* sp.**	Cryptinae		[22]	[22]				
***Gelis acarorum*** (Linnaeus, 1758)	Cryptinae	[31] (*Gelis sericeus*), [3, 4, 5, 24, 26, 28, 30] (*Pezomachus sericeus*)		[31] (*Gelis sericeus*), [3, 4, 5, 24, 26, 30] (*Pezomachus sericeus*)				
***Gelis areator*** (Panzer, 1804)	Cryptinae	[3, 4, 5, 11, 26, 28, 29, 30] (*Hemiteles areator*), [29, 30] (*Hemiteles pulchellus*)	[6, 16, 18, 19, 20]	[3, 4, 5, 26, 29, 30] (*Hemiteles areator*), [29, 30] (*Hemiteles pulchellus*), [6, 16, 18, 19, 20]				
***Gelis cinctus*** (Linnaeus, 1758)	Cryptinae		[16, 17, 18, 19, 20, 32]	[16, 17, 18, 19, 20, 32]				
• *Gelis sericeus* (Förster, 1850) = syn. *Gelis acarorum* (Linnaeus, 1758)								
• *Habrocryptus alternator* (Gravenhorst, 1829) = syn. *Ischnus alternator* (Gravenhorst, 1829)								
• *Habrocryptus punctiger* Thomson, 1896 = syn. *Ischnus migrator* (Fabricius, 1775)								
• *Habrocyptus punctiger* = misspelling for *Habrocryptus punctiger* Thomson, 1896								
• *Habrocryptus puntiger* = misspelling for *Habrocryptus punctiger* Thomson, 1896								
• *Helcostizus nigrocinctus* (Gravenhorst, 1815) = syn. *Aptesis nigrocincta* (Gravenhorst, 1815)								
***Hemiteles* sp.**	Cryptinae	[8, 13]	[9]	[13]				[9]
• *Hemiteles areator* (Panzer, 1804) =syn. *Gelis areator* (Panzer, 1804)								
• *Hemiteles hemipterum* (Fabricius, 1793) = misspelling for *Hemiteles hemipterus* (Fabricius, 1793)								
• *Hemiteles hemipterus* (Fabricius, 1793) = syn. *Theroscopus hemipteron* (Riche, 1791)								
• *Hemiteles nigriventris* Thomson, 1884 = syn. *Isadelphus gallicola* (Bridgman, 1880)								
• *Hemiteles pulchellus* Gravenhorst, 1829 = syn. *Gelis areator* (Panzer, 1804)								
• *Hemiteles sordipes* Gravenhorst, 1829 = syn. *Mastrus sordipes* (Gravenhorst, 1829)								
• *Herpestomus furunculus* Wesmael, 1845 = syn. *Herpestomus nasutus* Wesmael, 1845								
***Herpestomus* sp.**	Ichneumoninae	[24, 30]		[24, 30]				
***Herpestomus nasutus*** Wesmael, 1845	Ichneumoninae	[2, 3, 4, 5, 11, 26, 28, 29, 30] (*H. furunculus*)		[3, 4, 5, 26, 29, 30] (*H. furunculus*)				
***Isadelphus* sp.**	Cryptinae		[23]	[23]				
***Isadelphus gallicola*** (Bridgman, 1880)	Cryptinae	[26, 29, 30] (*Hemiteles nigriventris*)		[26, 29, 30] (*Hemiteles nigriventris*)				
***Ischnus* sp.**	Cryptinae		[6]	[6]				
***Ischnus alternator*** (Gravenhorst, 1829)	Cryptinae	[2, 4, 5, 26, 29, 30, 31] (*Habrocryptus alternator*)	[1, 13, 16, 17, 18, 19, 20, 22, 32]	[4, 5, 26, 29, 30, 31] (*Habrocryptus alternator*), [13, 16, 17, 18, 19, 20, 22, 32]	[1]			
***Ischnus migrator*** (Fabricius, 1775)	Cryptinae	[2, 3, 31] (*Habrocytus punctiger*), [2, 11, 26, 29, 30, 31] (*Habrocryptus punctiger*), [4, 5] (*Habrocryptus puntiger*)		[3, 31] (*Habrocytus punctiger*), [26, 29, 30, 31] (*Habrocryptus punctiger*), [4, 5] (*Habrocryptus puntiger*)				
***Itoplectis* sp.**	Pimplinae		[11]	[11]				
***Itoplectis alternans*** (Gravenhorst, 1829)	Pimplinae	[2, 3, 4, 5, 11, 24, 26, 28, 29, 30, 31] (*Pimpla alternans*)	[6, 9, 16, 17, 18, 19, 20, 22, 23, 25]	[3, 4, 5, 24, 26, 28, 29, 30, 31] (*Pimpla alternans*), [6, 16, 17, 18, 19, 20, 22, 23]	[25]	[28, 30] (*Pimpla alternans*)		[9]
***Itoplectis maculator*** (Fabricius, 1775)	Pimplinae	[2, 4, 5, 26, 29, 30] (*Pimpla maculator*)	[1]	[4, 5, 26, 29, 30] (*Pimpla maculator*)	[1]			
***Itoplectis tunetana*** (Schmiedeknecht, 1914)	Pimplinae		[16, 17, 18, 19, 20, 22, 25, 32]	[16, 17, 18, 19, 20, 22, 32]	[25]			
***Linycus exhortator*** (Fabricius, 1787)	Ichneumoninae	[26, 29, 30] (*Platylabus dimidiatus*)		[26, 29, 30] (*Platylabus dimidiatus*)				
***Mastrus sordipes*** (Gravenhorst, 1829)	Cryptinae	[2, 4, 5, 26, 29, 30] (*Hemiteles sordipes*)		[4, 5, 26, 29, 30] (*Hemiteles sordipes*)				
• *Microcryptus nigrocinctus* (Gravenhorst, 1815) = syn. *Aptesis nigrocincta* (Gravenhorst, 1815)								
• *Microcryptus nigrolineatus* = misspelling for *Microcryptus nigrocinctus* (Gravenhorst, 1815)								
• *Microcryptus nigrotinctus* = misspelling for *Microcryptus nigrocinctus* (Gravenhorst, 1815)								
***Nemeritis* sp.**	Campopleginae	[8]						
• *Omorgus difformis* (Gmelin, 1790) = syn. *Campoplex difformis* (Gmelin, 1790)								
• *Omurgus difformis* = misspelling for *Omorgus difformis* (Gmelin, 1790)								
***Parania geniculata*** (Holmgren, 1857)	Anomaloninae	[28, 30] (*Atrometus geniculatus*)		[28, 30] (*Atrometus geniculatus*)				
• *Pezomachus sericeus* Förster, 1850 = syn. *Gelis acarorum* (Linnaeus, 1758)								
***Phaeogenes* sp.**	Ichneumoninae	[2, 4, 5, 26, 29, 30]		[4, 5, 26, 29, 30]				
• *Phytodiaetus* sp. = misspelling for *Phytodietus* sp.								
***Phytodietus* sp.**	Tryphoninae		[1, 16, 18, 19, 20] (*Phytodiaetus* sp.)	[16, 18, 19, 20] (*Phytodiaetus* sp.)	[1] (*Phytodiaetus* sp.)			
• *Phytodietus pleuralis* Cresson, 1865 = North-American species, errouneously reported for Italy in Leonardi (1925), after Silvestri (1912)								
***Phytodietus polyzonias*** (Förster, 1771)	Tryphoninae		[6], [15] (*Phytodietus segmentator*)	[6]				[15] (*Phytodietus segmentator*)
• *Phytodietus segmentator* Gravenhorst, 1829 = syn. *Phytodietus polyzonias* (Förster, 1771)								
***Pimpla* sp.**	Pimplinae	[13]		[13]				
• *Pimpla alternans* Gravenhorst, 1829 = syn. *Itoplectis alternans* (Gravenhorst, 1829)								
***Pimpla apricaria*** Costa, 1885	Pimplinae		[9]					[9]
• *Pimpla detrita* Holmgren, 1860 = syn. *Endromopoda detrita* (Holmgren, 1860)								
• *Pimpla examinator* (Fabricius, 1804) = syn. *Pimpla turionellae* (Linnaeus, 1758)								
• *Pimpla maculator* (Fabricius, 1775) = syn. *Itoplectis maculator* (Fabricius, 1775)								
***Pimpla spuria*** Gravenhorst, 1829	Pimplinae	[2, 3, 4, 5, 11, 26, 28, 29, 30, 31] (*P. strigipleuris*)	[1, 6, 16, 17, 18, 19, 20, 22, 23]	[6, 16, 17, 18, 19, 20, 22, 23], [3, 4, 5, 26, 29, 30, 31] (*P. strigipleuris*)	[1]			
• *Pimpla strigipleuris* Thomson, 1877 = syn. *Pimpla spuria* Gravenhorst, 1829								
***Pimpla turionellae*** (Linnaeus, 1758)	Pimplinae	[2, 4, 5, 8, 11, 26, 28, 29, 30, 31], [2, 4, 5, 26, 28, 29, 30] (*P. examinator*)	[9, 16, 18, 19, 20, 32]	[4, 5, 16, 18, 19, 20, 26, 28, 29, 30, 31, 32], [4, 5, 26, 28, 29, 30] (*P. examinator*)		[28, 30]		[9]
• *Platylabus dimidiatus* (Gravenhorst, 1829) = syn. *Lynicus exhortator* (Fabricius, 1787)								
***Pristomerus vulnerator*** (Panzer, 1799)	Cremastinae		[9, 16, 18, 19, 20]	[16, 18, 19, 20]				[9]
***Scambus elegans*** (Woldstedt, 1877)	Pimplinae		[9]					[9]
***Sinophorus crassifemur*** (Thomson, 1887)	Campopleginae	[29, 30] (*Eulimneria crassifemur*) [26, 29] (*Eulimneria ramifemur*)		[29, 30] (*Eulimneria crassifemur*) [26, 29] (*Eulimneria ramifemur*)				
***Sinophorus turionum*** (Ratzeburg, 1844)	Campopleginae	[31] (*Eulimneria alkae*)						
• *Spilocryptus* sp. = syn. *Agrothereutes* sp.								
***Theroscopus hemipteron*** (Riche, 1791)	Cryptinae	[26] (*Hemiteles hemipterum*), [2, 4, 5, 29, 30] (*Hemiteles hemipterus*)	[1, 9, 16, 18, 19, 20, 32] (*T. hemipterus*)	[26] (*Hemiteles hemipterum*), [4, 5, 29, 30] (*Hemiteles hemipterus*), [16, 18, 19, 20, 32] (*T. hemipterus*)	[1] (*T. hemipterus*)			[9] (*T. hemipterus*)
• *Theroscopus hemipterus* (Fabricius, 1793) = syn. *Theroscopus hemipteron* (Riche, 1791)								
• *Thyraeella collaris* (Gravenhorst, 1829) = syn. *Diadromus collaris* (Gravenhorst, 1829)								
• *Thyrella collaris* = misspelling for *Thyraeella collaris* (Gravenhorst, 1829)								
***Tranosemella praerogator*** (Linnaeus, 1758)	Campopleginae		[6, 7, 16, 18, 19, 20]	[6, 7, 16, 18, 19, 20]				
***Trichomma enecator*** (Rossi, 1790)	Anomaloninae		[6, 25]	[6]	[25]			
***Triclistus* sp.**	Metopiinae		[1, 15, 17, 23]	[17, 23]	[1]			[15]
***Triclistus albicinctus*** Thomson, 1887	Metopiinae		[16, 18, 19, 20]	[16, 18, 19, 20]				
***Triclistus lativentris*** Thomson, 1887	Metopiinae		[9]					[9]
• *Triclistus nitidifrons* = misspelling for *Triclistus nitifrons* Thomson, 1887								
• *Triclistus nitifrons* Thomson, 1887 = syn. *Triclistus pallipes* Holmgren, 1873								
• *Triclistus pallidipes* = misspelling for *Triclistus pallipes* Holmgren, 1873								
***Triclistus pallipes*** Holmgren, 1873	Metopiinae	[28] (*T. nidifrons*), [8, 11] (*T. nitidifrons*), [28, 30] (*T. pallidipes*)	[25]		[25]	[28, 30] (*T. pallidipes*)		
***Venturia canescens*** (Gravenhorst, 1829)	Campopleginae		[16, 18, 19, 20]	[16, 18, 19, 20]				

* see text about the misidentification of *Campoplex capitator* and *C. difformis* in the works of Catoni (1910, 1914) and Silvestri (1912), then reported later also by Leonardi (1925) and Boselli (1928).

In the present work, we focus on the ichneumonid subfamilies Anomaloninae and Campopleginae. So far, four taxa of Anomaloninae and 27 of Campopleginae have been associated with EGVM (Table 3).

**Table 3.** Ichneumonidae Anomaloninae and Campopleginae reported by various authors as living at the expense of the European grapevine moth.

	Valid names	Name reported by the author/s	Author/s and year of publication	Country
ANOMALONINAE				
1	***Agrypon anxium*** (Wesmael,1849)	***Agrypon anxium*** (Wesmael,1849)	Moreau et al. 2010	France, Switzerland
2	***Agrypon flaveolatum*** (Gravenhorst, 1807)	*Agrypon flaveolatum*	Schwangart 1913, 1918	Germany (Palatinate), Italy (South Tyrol)
		Agrypon (Anomalon) flaveolatum	Feytaud 1913	Germany
		*Agrypon flaveolatum* Grav.	Stellwaag 1921	Germany (Franconia and Rhineland- Palatinate), Italy (South Tyrol)
		*Agrypon flaveolatum* Grav.	Stellwaag 1928	
		*Agrypon flaveolatum* Grav.	Thompson 1946	Austria, France, Germany
3	***Parania geniculata*** (Holmgren, 1857)	*Atrometus geniculatus* Forst.	Silvestri 1912	Italy (South Tyrol)
		*Atrometus geniculatus* Först.	Stellwaag 1928	Italy (South Tyrol)
		*Sinophorus geniculatus* Grav.	Hoffmann and Michl 2003	
4	***Trichomma enecator*** (Rossi, 1790)	*Trichomma enecator* (Rossi 1790)	Telenga 1934	Crimea
		*Trichomma enecator* (Rossi 1790)	Colombera et al. 2001	Italy (Piedmont)
		*Trichomma enecator*	Scaramozzino et al. 2017b	Italy (Tuscany)
CAMPOPLEGINAE				
**1**	***Campoplex abbreviatus*** (Brischke, 1880)	Mesoleius (Omorgus) abbreviatus Brischke	Stellwaag 1928 (after Schwangart)	
		*Campoplex abbreviatus* Brischke	Thompson 1946	France
		*Campoplex abbreviatus* Brisch.	Hoffmann and Michl 2003	
**2**	***Campoplex borealis*** (Zetterstedt, 1838)	*Campoplex borealis*	CABI 2017	
**3**	***Campoplex capitator*** Aubert, 1960	*Campoplex capitator*	CABI 2017	
		*Campoplex capitator* Aubert	Hoffmann and Michl 2003	
		*Campoplex capitator* Aub.	Koclu et al. 2005	Turkey
		*Campoplex capitator* Aubert,1960	Villemant et al. 2011	France, Italy, Portugal, Spain
		*Campoplex capitator* Aubert,1960	Yu et al. 2012	
**4**	***Campoplex difformis*** (Gmelin, 1790)	*Omorgus difformis* (Gmel.) Thoms	Stellwaag 1928 (after Rübsaamen, Marchal, Catoni, Schwangart, Feytaud, Silvestri, Ruschka and Fulmek, Dobredev, Voukassovitch)	Italy (South Tyrol),Austria
		*Omorgus difformis* Gm.	Telenga 1934	Crimea
		*Campoplex difformis* Grav.	Thompson 1946 (after Telenga)	Austria, France, Germany, Italy, Crimea
**4**	***Campoplex difformis*** (Gmelin, 1790)	*Campoplex difformis* Gmelin	Hoffmann and Michl 2003	
		*Campoplex difformis*	CABI 2017	
		*Campoplex difformis* (Gmelin,1790)	Yu et al. 2012	
**5**	***Campoplex* sp.**	*Campoplex* sp.	Thompson 1946	France
**6**	***Diadegma areolare*** (Holmgren, 1860)	*Angitia areolaris* (Holmgr.) Thoms.	Stellwaag 1928 (after Rübsaamen, Schwangart, Dobredev)	
		*Angitia areolaris* Hlgr.	Thompson 1946	Russia
**7**	***Diadegma armillatum*** (Gravenhorst, 1829)	Angitia (Dioctes) tibialis Grav.	Stellwaag 1928 (after Silvestri)	Italy
		*Diadegma armillatum* Grav.	Hoffmann and Michl 2003	
**8**	***Diadegma fenestrale*** (Holmgren, 1860)	*Angitia fenestralis* (Holmgr.) Thoms.	Stellwaag 1928(after Rübsaamen,Schwangart,Dobredev)	
		*Angitia fenestralis* Hlgr.	Thompson 1946	Russia
		*Diadegma fenestrale* Holm.	Hoffmann and Michl 2003	
		*Diadegma fenestrale* (Holmgren,1860)	Villemant et al. 2011	France, Germany, Switzerland
**9**	***Diadegma majale*** (Gravenhorst,1829)		Marchal 1912, Feytaud 1913	France
**10**	***Diadegma melanium*** (Thomson, 1887)	*Angitia melania* Thoms.	Stellwaag 1928 (after Marchal)	France (envir. Paris)
		*Angitia melania* Thoms.	Thompson 1946	Russia
		*Diadegma melanium* Thoms.	Hoffmann and Michl 2003	
**11**	***Diadegma holopygum*** (Thomson, 1887)	*Diadegma holopygum* (Thomson 1887)	Yu et al. 2012	
		*Diadegma holopyga* (Thoms.)	Bărbuceanu and Jenser 2009	Romania (South)
**12**	***Diadegma longicaudatum*** Horstmann, 1969	*Diadegma longicaudatum* Horstmann 1969	Yu et al. 2012	
		*Diadegma longicaudata* Horst.	Bărbuceanu and Jenser 2009	Romania (South)
**13**	***Diadegma* sp.**	*Angitia* sp.	Thompson 1946	France
**14**	***Diadegma tenuipes*** (Thomson, 1887)	*Angitia tenuipes* Thoms.	Stellwaag 1928 (after Catoni, Schwangart)	Italy (South Tyrol)
		*Angitia tenuipes* Thoms.	Thompson 1946	Italy
		*Diadegma tenuipes* Thoms.	Hoffmann and Michl 2003	
		*Diadegma tenuipes* (Thomson 1887)	Yu et al. 2012	
		*Diadegma tenuipes* (Thoms.)	Bărbuceanu and Jenser 2009	Romania (South)
**15**	***Diadegma trochanteratum*** (Thomson, 1887)	*Angitia trochanterata* Thoms.	Stellwaag 1928(after Schwangart)	
		*Diadegma trochanteratum* Thoms.	Hoffmann and Michl 2003	
**16**	***Enytus apostata*** (Gravenhorst, 1829).	*Angitia exareolata* (Ratzebg.) Thoms.	Stellwaag 1928 (after Rübsaamen, Marchal, Dobredev)	
		*Diadegma exareolatus*	Feytaud 1913 (after Marchal)	France
		*Angitia exareolata* Ratz.	Thompson 1946	Russia
**16**	***Enytus apostata*** (Gravenhorst, 1829).	*Enytus apostatus* Grav.	Hoffmann and Michl 2003	
		*Enytus apostatus* (Gravenhorst 1829)	Yu et al. 2012	
		*Enytus apostata* Gravenhorst	Lotfalizadeh et al. 2012	Iran
		*Diadegma apostata* (Grav.)	Bărbuceanu and Jenser 2009	Romania (South)
**17**	***Enytus obliteratus*** (Cresson, 1864)	*Enytus obliteratus* (Cresson 1864)	Yu et al. 2012	
**18**	***Hyposoter ebeninus*** (Gravenhorst, 1829)	*Anilastus ebeninus* Grav.	Telenga 1934	Crimea
		*Anilastus ebeninus* Grav.	Thompson 1946	Russia
		*Hyposoter ebeninus* (Gravenhorst 1829)	Yu et al. 2012	
**19**	***Lathrostizus lugens*** (Gravenhorst, 1829)	*Angitia vestigialis* (Ratzbg.) Thoms	Stellwaag 1928 (after Rübsaamen, Marchal, Feytaud, Dobredev)	
		*Angitia vestigialis*, Ratz.	Marchal 1912	France
		*Diadegma vestigialis*	Feytaud 1913 (after Marchal)	France
		*Angitia vestigialis* Ratz.	Thompson 1946	Russia
		*Lathrostizus lugens* Grav.	Hoffmann and Michl 2003	
**20**	***Meloboris collector*** (Thunberg, 1822)	*Nepiera collector* Thnbg.	Thompson 1946	France
		*Nepiera concinna* Hlgr.	Faure 1925	France
		*Meloboris collector* Thunberg	Hoffmann and Michl 2003	
		*Meloboris collector* (Thunberg 1822)	Yu et al. 2012	
	[***Olesicampe argentata*** (Gravenhorst, 1829)]	*Olesicampe argentata* Grav.	Hoffmann and Michl 2003	Wrong record based on incorrect synonymy
**21**	***Olesicampe* sp.**	*Limneria* spec.	Feytaud 1913	France
		*Olesicampe* Förster	Hoffmann and Michl 2003	
**22**	***Sinophorus costalis*** (Thomson, 1887)	*Sinophorus costalis* Thoms.	Pisicá and Páişescu-Bărbuceanu 2002	Romania
**23**	***Sinophorus crassifemur*** (Thomson, 1887)	*Sinophorus crassifemur* (Thomson 1887)	Yu et al. 2012	
	[***Sinophorus geniculatus*** (Gravenhorst, 1829)]	*Sinophorus geniculatus* Grav.	Hoffmann and Michl 2003	Wrong record based on incorrect synonymy
**24**	***Sinophorus turionum*** (Ratzeburg, 1844)	*Eulimneria alkae* E. and S.	Thompson 1946	Austria, France, Germany, Italy
		*Sinophorus turionum* Ratzeburg	Hoffmann and Michl 2003	
		*Campoplex alkae*	CABI 2017	
		*Sinophorus turionum* (Ratzeburg 1844)	Yu et al. 2012	
**25**	***Tranosemella praerogator*** (Linnaeus, 1758)	*Tranosemella praerogator*	CABI 2017	
		*Tranosemella praerogator* Linn.	Hoffmann and Michl 2003	
		*Tranosemella praerogator* (Linnaeus 1758)	Villemant et al. 2011	France, Italy
		*Tranosemella praerogator* (Linnaeus 1758)	Yu et al. 2012	
**26**	***Venturia canescens*** (Gravenhorst, 1829)	*Venturia canescens* Grav.	Hoffmann and Michl 2003	
		*Venturia canescens* (Gravenhorst 1829)	Thiéry et al. 2001, Villemant et al. 2011	France, Italy
		*Venturia canescens* (Gravenhorst 1829)	Yu et al. 2012	
**27**	***Venturia* sp.**	*Venturia* sp.	Koclu et al. 2005	Turkey

### Order: HYMENOPTERA

#### Superfamily: ICHNEUMONOIDEA

##### Family: ICHNEUMONIDAE

###### Subfamily: Anomaloninae

####### Agrypon flaveolatum

Taxon classificationAnimaliaHymenopteraIchneumonidae

(Gravenhorst, 1807)

Agrypon flaveolatum : Schwangart 1913: 6, 1918: 547.
Agrypon flaveolatum : Stellwaag 1921: 85-86.

######## Italian distribution of reared parasitoids.

Trentino-South Tyrol: Schwangart 1913, 1918; Stellwaag 1921.

######## Distribution.

Species of temperate-cold zones of the Palearctic region, widespread in Europe (excluding the Balkan Peninsula), Russia, Turkey, eastwards to Korea and Japan (Yu et al. 2012). From Europe it has been introduced in Canada from 1956 to 1958 and from 1979 to 1981 to control the winter moth *Opheroptera brumata* (Linnaeus, 1758) (Lepidoptera, Geometridae) and has been reported as being established there (Carlson 1979, Barron 1989).

######## Host range.

Yu et al. (2012) list 58 species, mostly belonging to the Lepidopteran family Geometridae (27 species). Further records include Tortricidae (8 species), Yponomeutidae (7), Noctuidae (5), Lasiocampidae, Nolidae (2 species each), Pyralidae, Thyatiridae, Notodontidae, Lycaenidae, Erebidae (*Lymantria dispar* (Linnaeus, 1758)), and Diprionidae (Hymenoptera Symphyta) (1 species each).

######## Ecological role.

This larval-pupal koinobiont endoparasitoid emerged from overwintering pupae of *L. botrana* and *Eupoecilia ambiguella* (Hübner, 1796) (Stellwaag 1928, Thompson 1957) in Italy (South Tyrol), France and Germany and *Sparganothis pilleriana* (Denis & Schiffermüller, 1775) (Lepidoptera Tortricidae) in France (Stellwaag 1928). These hosts are not included in the list of Yu et al. (2012). Another species of the same genus, *Agrypon anxium* (Wesmael, 1849), is reported on the first generation larvae of EGVM in Switzerland and France (Moreau et al. 2010), while *A. minutum* (Bridgman and Fitch, 1894) has been obtained by *E. ambiguella* in France and Switzerland and by *S. pilleriana* in Germany (Villemant et al. 2011).

######## Taxonomic notes.

After its introduction in Canada, *A. flaveolatum* was confused by some American authors (Carlson 1979, Dasch 1984) with similar Nearctic species belonging to the same genus (Barron 1989).

####### Parania geniculata

Taxon classificationAnimaliaHymenopteraIchneumonidae

(Holmgren, 1857)

Figure 1

Atrometus geniculatus : Silvestri 1912: 296; Stellwaag 1928: 665.
Sinophorus geniculatus : Hoffmann and Michl 2003: 3 (misinterpretation).

######## Italian distribution of reared parasitoids.

Trentino-South Tyrol: Silvestri 1912.

######## Distribution.

This species is widespread over most of the temperate Holarctic region. It is quite common in the Nearctics (Yu et al. 2012), while in the Palaearctics its distribution is limited to the Western part only: Europe, Central Russia and Turkey (Yu et al. 2012, Zwakhals and van Achterberg 2017).

######## Host range.

Yu et al. (2012) list 38 hosts for this species, 21 of which belonging to the family Tortricidae, the remaining, in order of importance, to the families Pyralidae (4 species), Gelechiidae (3), Noctuidae (2), Choreutidae, Galacticidae, Geometridae, Lycaenidae, Nolidae, Sesiidae and Psychidae (all represented by a single species). Some economically important species are included, as the Oriental fruit moth *Grapholita molesta* (Busck, 1916) and the codling moth *Cydia pomonella* (Linnaeus, 1758), *Argyrotaenia* spp., *Rhyacionia* spp., *Choristoneura* spp. (Tortricidae), and the European corn borer, *Ostrinia nubilalis* (Hübner, 1796) (Crambidae). The report of *Andricus kollari* (Hartig, 1843) (Hymenoptera Cynipidae) of Fulmek (1968) as host of *P. geniculata* is not reliable.

In Italy, the species is reported on *Paranthrene tabaniformis* (Rottemburg, 1775) (Lepidoptera Sesiidae) (Dasch 1984, without indication of locality), on *Gypsonoma aceriana* (Duponchel, 1843) (Lepidoptera Tortricidae) (Haeselbarth 1989) and *L. botrana* (Silvestri 1912). The latter is not included in the list of Yu et al. (2012) and represents the only record for this species on EGVM.

######## Ecological role.

Silvestri (1912) reared a single male of this species from overwintering pupae of EGVM in San Michele (Trentino) in May.

######## Taxonomic notes.

**Figure 1.**
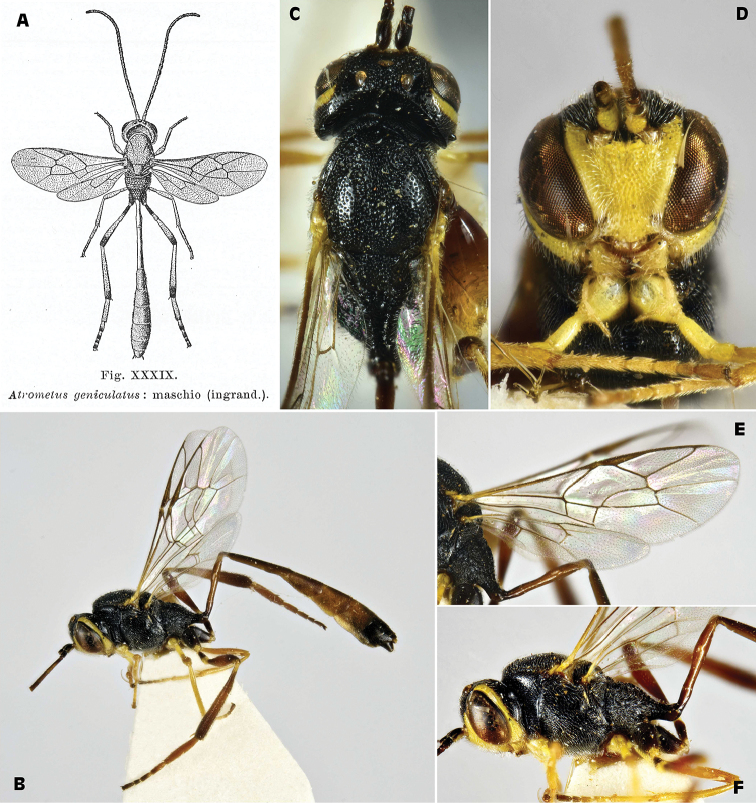
*Parania geniculata* (Holmgren, 1857), male (IT, Udine, Aug. 28, 1985, leg. Allegro ex *Gypsonoma aceriana*; ZSM) **A** drawing of male in a dorsal vision by Silvestri (1912) **B** habitus, lateral view **C** head and anterior part of thorax, dorsal view **D** head, frontal view **E** wings **F** head and mesosoma lateral view.

*Parania geniculata* is one of the smallest European species of the subfamily Anomaloninae. It parasitizes mostly Tortricidae (Schnee 2008). The species was originally described by Holmgren (1857) as *Anomalon geniculatum* and subsequently transferred to the genus *Atrometus* Förster, 1869 by Thomson (1892). Silvestri (1912) has erroneously attributed the authorship of the species to Förster, who just described the genus *Atrometus* (Förster 1869). Then Townes (1971) transferred the species to *Parania* Morley, 1913, with *P. geniculata* as the only European species of this small, but widely distributed genus. Probably because of this nomenclatural inexactness, Hoffmann and Michl (2003) have misinterpreted the specimen obtained by Silvestri and put it in synonymy with *Sinophorus geniculatus* Gravenhorst, 1829, which belongs to the subfamily Campopleginae. The specimen figured by Silvestri (1912, fig. XXXIX, Figure 1A) clearly belongs to Anomaloninae. After comparing the figure by Silvestri with specimens of *Atrometus insignis* Förster, 1878 (a South European species that could be confused with *Parania*), and *Parania geniculata* in his collection, Heinz Schnee recognized the specimens depicted by Silvestri as *P. geniculata*, for the following reasons: “…small number of flagellomeres, small brachial cell, mesoscutum and scutellum somewhat longer, and slender hind tarsi”; on the contrary, “…*Atrometus insignis* is therefore out of the question, because in the drawing the characteristic transverse furrow on posterior part of the mesoscutum is absent and the brachial cell is too small. Also the hind tarsi of *P. geniculata* are much thinned, while they are strongly thickened in males of *A. insignis*. Moreover, the hosts of *A. insignis* are *Zygaena* spp. (Lepidoptera Zygaenidae) and other host assignments are very likely wrong (Schnee in litteris)”. We have searched for the specimen identified by Silvestri without finding it.

####### Trichomma enecator

Taxon classificationAnimaliaHymenopteraIchneumonidae

(Rossi, 1790)

Figure 2

Trichomma enecator : Colombera et al. 2001: 94; Scaramozzino et al. 2017b: 133.

######## Italian distribution of reared parasitoids.

Piedmont: Colombera et al. 2001.

Tuscany: Scaramozzino et al. 2017b.

######## Distribution.

Palaearctic species occurring throughout Europe, Canary Islands, Near East (Turkey and Syria), Northern and Central Russia, Kazakhstan, Russian Far East, Korea and Japan (Yu et al. 2012; Zwakhals and van Achterberg 2017). In 1931, it was released in North America (New Jersey, USA) to control the Oriental fruit moth, without becoming established (Carlson 1979).

######## Host range.

Females lay eggs on young larvae that live hidden in the vegetation. Yu et al. (2012) list 28 host species, many of which belong to the family Tortricidae, including the fruit crop pests *Cydia pomonella* (Linnaeus, 1758), *Archips rosana* (Linnaeus, 1758) and *Grapholita molesta* (Busck, 1916). The following species need to be added to the list: Aphelia (Zelotherses) peramplana (Hübner, 1825) (= *A. amplana* Hübner, 1813) (Vas et al. 2015), *Archips crataegana* (Hübner, 1799) (Lungu-Constantineanu 2009), *Cacoecimorpha pronubana* (Hübner, 1799) (Scaramozzino et al. 2017b), *Cydia pactolana* (Zeller, 1840) (Jansons 2013), *Eudemis profundana* (Denis & Schiffermüller, 1775) (Zaemdzhikova 2017), *Sparganothis pilleriana* (Denis & Schiffermüller, 1775) (Habermehl 1922), *Spilonota ocellana* (Denis & Schiffermüller, 1775) (Nuzhna 2010) among the Tortricidae; *Anacampsis populella* (Clerck, 1759) (Hassanein 1978) among Gelechiidae; *Macrothylacia rubi* (Linnaeus, 1758) (Nuzhna 2010) among Lasiocampidae; *Acrobasis suavella* (Zincken, 1818) (Morley 1915) among Pyralidae; and *Prismosticta fenestrata* Butler, 1880 among Endromidae (Morley 1915). Habermehl (1922) reports as host also *Gelechia boticella*, but the identity of this species still remains unclear.

Ultimately, 40 host species of *Trichomma enecator* have been reported: 25 species belonging to Tortricidae, three each to Gelechiidae and Pyralidae, and one each to Elachistidae, Endromidae, Erebidae (*Lymantria dispar* (Linnaeus, 1758)), Gracillariidae, Lasiocampidae, Noctuidae, Nolidae and Psychidae. The record by Starke (1956 in Yu et al. 2012) of *Plioreocepta poeciloptera* (Schrank, 1776) (Diptera Tephritidae) seems unlikely and is probably wrong.

In Italy, *T. enecator* is reported on: *Earias clorana* (Linnaeus, 1761) (Lepidoptera Nolidae) on goat willow (*Salix caprea* Linnaeus, 1753) (Leonardi 1925); *Cydia pomonella* in Campania (Sciarra 1915) and Emilia Romagna (Faggioli 1938); *Tortrix viridana* Linnaeus, 1758 and *Aleimma loeflingiana* (Linnaeus, 1758) (Lepidoptera Tortricidae) on oak in Calabria and Campania, respectively (Silvestri 1923); *Grapholita molesta* in Emilia Romagna (Grandi 1937); *Rhyacionia buoliana* (Denis & Schiffermüller, 1775) in Tuscany (Zocchi 1952); *Cacoecimorpha pronubana* feeding on spurge flax (*Daphne gnidium* Linnaeus, 1753) in Tuscany (Scaramozzino et al. 2017b).

######## Ecological role.

*Trichomma enecator* is a solitary, koinobiont, larval-pupal endoparasitoid on fruit-mining or other concealed lepidopterous larvae. It is one of the most common parasitoids of the codling moth in Europe (Franck et al. 2017). Although quite common, its control action on the codling moth is limited, with parasitization rates rarely exceeding 5% (Table 4), being inexplicably absent in some apple orchards (Maalouly et al. 2013).

**Table 4.** *Trichomma enecator* (Rossi): parasitization rates recorded on *Cydia pomonella* and other hosts.

Species	Place	Parasitization rate	Reference	Notes
*Cydia pomonella*	France	0.23–3.11	Rosenberg (1934)	Winter generation on apple; found in 8 out of 11 locations studied
*Cydia pomonella*	Switzerland, South-West	0.6–2.9	Athanassov et al. (1998)	Winter generation on apple; *T. enecator* represents 42% of all the reared parasitoids
*Cydia pomonella*	Syria, coastal region	5.64	Ismail and Albittar (2016)	In a neglected apple orchards in 2003
*Cydia pomonella*	Syria, coastal region	1.92–3.27	Ismail and Albittar (2016)	In two neglected apple orchards in 2004, all generations
*Cydia pomonella*	Germany, Baden-Württemberg	1.22	Lashkari-Bod and Zebitz (2014)	On apple, from spring and autumn collections
*Cydia pomonella*	Switzerland, South	9.9	Mills (2005)	Maximum percent rates recorded in an individual orchard
*Cydia pomonella*	Austria, East	7.4	Mills (2005)	Maximum percent rates recorded in an individual orchard
*Cydia pomonella*	Spain, Asturia	0.26–0.83	Miñarro and Dapena (2004)	In 2001 and 2002 respectively; winter generation on apple
*Cydia pomonella*	Switzerland, upper Rhine-valley	parasitization rates as in Wildbolz and Staub (1985) and Athanassov et al. (1998)	Höhn et al. (1999)	Winter generation on apple
*Archips crataegana*	Romania, Iaşy	9.21	Lungu-Constantineanu (2009)	From oak (*Quercus petraea*)
*Archips rosana*	Poland, environs of Poznań	0.48–0.28 (average of two years = 0.76)	Piekarska-Boniecka (1994)	On red currant in 1989 and 1990 respectively

What we know about its biology is mainly due to Rosenberg (1934), who studied the codling moth in French apple orchards. This parasitoid attacks the host larvae, hibernating in the larval stage inside the host; the adult emerges from the pupa, some weeks before the host; the emergence period, in outdoor insectary, lasts 10–24 days, from the second half of May to the beginning of June. In captivity both genders may live approximately a month. Females start to oviposit one or two days after emergence, and their eggs hatch in approximately eight days (Rosenberg 1934). Despite its presence in most of the areas in Rosenberg’s survey, the parasitism rate never surpassed 3.11%.

*Trichomma enecator* females parasitize all the larval instars of the codling moth inside the fruits. The females are attracted by exudates that accumulate on the surface of the fruits infested by the codling moth larvae; in the absence of these exudates, the parasitization behavior is disrupted (Mills and Dixon 1993). To breed this species in insectarium is very difficult (Mills 2005), even if Russ and Faber (1978) were able to rear it until F6 generation by the same method used to rear *Ascogaster quadridentatus* Wesmael, 1845 (Hymenoptera, Braconidae).

At our latitude, *T. enecator* is a multivoltine species, while in Central-Northern Europe (Gauld and Mitchell 1977, Sedivy 2001) and Spain (Miñarro and Dapena 2004) it is presumably bivoltine. According to our personal observations, in Italy it shows three generations per year, and can attack all three generations of EGVM.

*Trichomma enecator* has a secondary importance on EGVM; Telenga (1934) obtained it in early June in Crimea. In Piedmont (Italy), a single specimen emerged from pupae of the overwintering generation of *L. botrana* (Colombera et al. 2001). In the Natural Reserve of San Rossore (Pisa, Tuscany), we obtained 13 specimens of *T. enecator* from EGVM pupae in July 2012 and from EGVM and *C. pronubana* pupae in May and July 2014. Pupae of both tortricids were collected into the nests formed by the larva on the apical buds of the spurge flax *Daphne gnidium* (Malvales, Thymelaeaceae) (Scaramozzino et al. 2017b).

**Figure 2.**
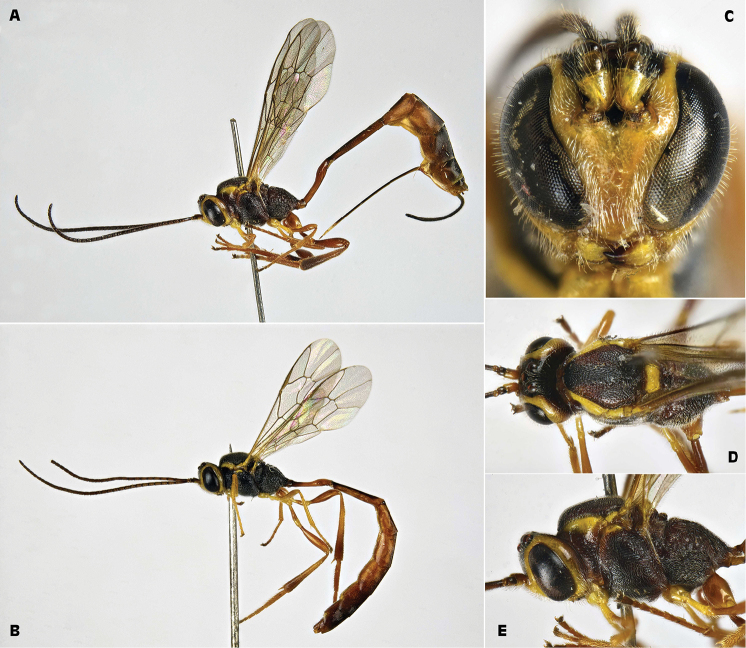
*Trichomma enecator* (Rossi, 1790) (IT, San Rossore Pisa, ex *Lobesia botrana*) **A** female habitus lateral view **B** male habitus lateral view **C** female head frontal view **D** female head and mesosoma dorsal view **E** female head and mesosoma lateral view.

###### Subfamily: Campopleginae

As mentioned, 27 taxa belonging to nine different genera of Campopleginae are reported on EGVM in Eurasia (Table 3). Many species of this subfamily are important natural enemies of insect pests (Lepidoptera above all) and were often used in biological control programs (Quicke 2015). The few species that have been studied in detail often represent the dominant component in parasitoid community of a given host and could be good biological control agent candidates (Jenner et al. 2005). Unfortunately, the uncertainty associated with the taxonomic status of many species and the lack of updated and well-illustrated literature often represent an obstacle to their use in biological control programs. Females are barely identifiable and males are often indeterminable (Horstmann 2012). Misidentifications are easy, making associated host ranges mostly unreliable (Fitton and Walker 1992, Shaw and Horstmann 1997, Jenner et al. 2013). It descends that it is difficult to adopt a species as a potential biological control agent, particularly focusing on the risk it could represent for non-target species in a new area. Probably, for many species the host range is narrower than that inferred from the literature (Jenner et al. 2013, Horstmann 2012).

####### Campoplex

Taxon classificationAnimaliaHymenopteraIchneumonidae

sp. Gravenhorst, 1829

Campoplex sp.: Pinna et al. 1989: 82; Lozzia and Rigamonti 1991: 34; Coscollá 1997: 218.

######## Italian distribution of reared parasitoids.

Piedmont: Pinna et al. 1989; Lozzia and Rigamonti 1991.

######## Distribution and host range.

The cosmopolitan genus *Campoplex* Gravenhorst, 1829 includes ca. 210 species of koinobiont endoparasitoids of microlepidopteran larvae (mainly of the Tortricidae family, but also of Coleophoridae, Gelechiidae, Pyralidae etc.), and to a lesser extent of macrolepidoptera, Hymenoptera Symphyta and few Coleoptera Curculionidae (see Yu et al. 2012 for a more comprehensive review). In Europe approximately 90 species are recorded (Zwakhals and van Achterberg 2017).

######## Ecological role.

Pinna et al. (1989) obtained an unidentified species of *Campoplex* from overwintering specimens of *L. botrana*, with parasitism rates variable from 4.7 to 36.9%. Lozzia and Rigamonti (1991) found an unidentified species from overwintering generation of the EGVM, with a parasitism rate close to 4%.

####### Campoplex borealis

Taxon classificationAnimaliaHymenopteraIchneumonidae

(Zetterstedt, 1838)

Figure 3

Campoplex borealis : Nuzzaci and Triggiani 1982: 49.

######## Italian distribution of reared parasitoids.

Apulia: Nuzzaci and Triggiani 1982 [on *Daphne gnidium* L.].

######## Distribution.

The species is widely spread throughout Europe and Northern Russia (Yu et al. 2012; Zwakhals and van Achterberg 2017). In 1937, 96 adults of *C. borealis* were released in U.S.A. (Connecticut) from Europe, to control the European pine shoot moth, *Rhyacionia buoliana* (Denis & Schiffermüller, 1775) (Lepidoptera Tortricidae), but the species has not become established (Dowden 1962, Bartlett et al. 1978).

######## Host range.

Yu et al. (2012) report 19 host species associated with *C. borealis*, belonging to the families Coleophoridae (8 species), Tortricidae (4 species, *L. botrana* is not into the list), Psychidae, Yponomeutidae, Eriocraniidae, Gelechiidae, Gracillariidae, Simaethidae (one species each) and on the weevil *Anthonomus pomorum* (Linnaeus, 1758) (Coleoptera Curculionidae).

######## Ecological role.

In three years of sampling on *Daphne gnidium*, Nuzzaci and Triggiani (1982) obtained three specimens of this parasitoid from larvae of *L. botrana* (identified by K. Horstmann). This is the only record on EGVM so far.

######## Taxonomic notes.

*Campoplex borealis* is the species that gives its name to a “*borealis*” species-group of the genus *Campoplex* (Horstmann 2012). With the name of *C. borealis* were indicated at least six different species (Horstmann 2012), morphologically very similar and mainly characterized by their host preferences. Right now, eight species are included in this species-group: *C. borealis* (Zetterstedt, 1838), *C. jaeckhi* (Bauer, 1936), *C. psammae* (Morley, 1915), *C. punctipleuris* Horstmann, 1980, *C. serratellae* Horstmann, 2012, *C. caloptiliae* Horstmann, 2013, *C. tussilaginis* Horstmann, 2013 and *Campoplex linosyridellae* Horstmann, 2016. They are mainly related to Coleophoridae and Gelechiidae; one species, *C. caloptiliae*, lives on Gracillariidae, while a second species close to *C. psammae* lives on Psychidae (Horstmann 1980, 2012, 2013, Shaw et al. 2016). Horstmann (1985) does not mention EGVM among the hosts of *C. borealis*, despite a male and a female collected by Nuzzaci and Triggiani are in his collection in ZSM.

**Figure 3.**
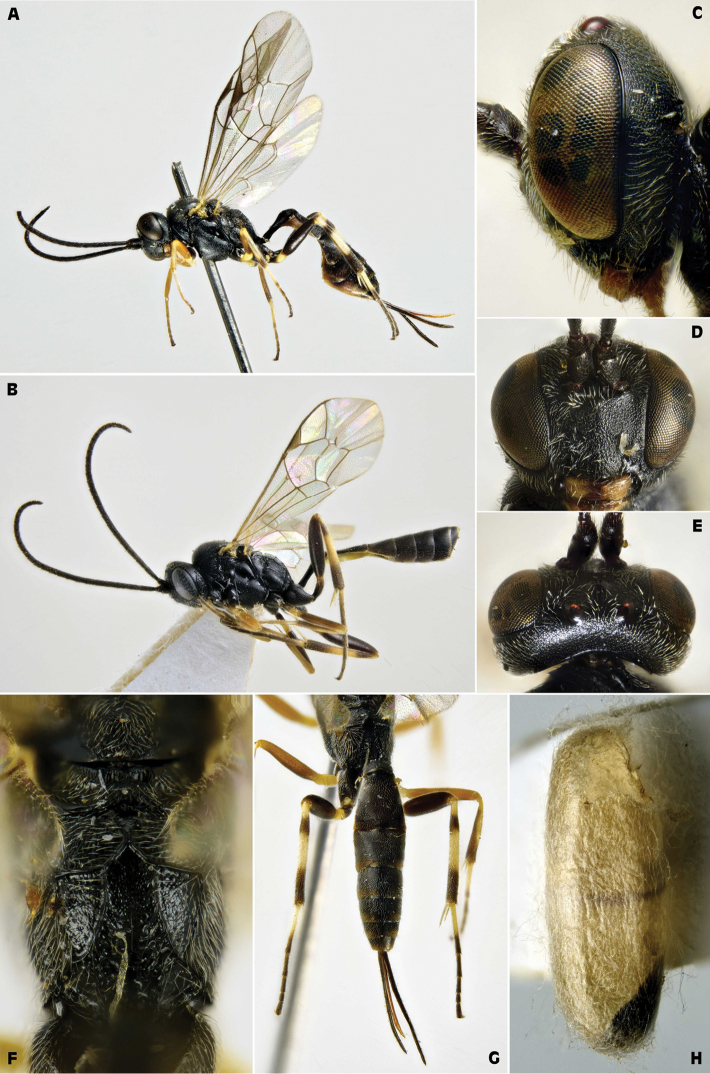
*Campoplex borealis* (Zetterstedt, 1838) (female: SP, Barcelona, March 28, 1964; male: CH, Sustenpaß, September 10, 1989, ex *Acleris variegana*; ZSM) **A** female habitus lateral view **B** male habitus lateral view **C** female head lateral view **D** female head frontal view **E** female head dorsal view **F** female propodeum dorsal view **G** female metasoma dorsal view **H** opened cocoon.

####### Campoplex capitator

Taxon classificationAnimaliaHymenopteraIchneumonidae

Aubert, 1960

Figures 4 , 5

Campoplex capitator : Marchesini and Dalla Montà 1992: 10, 1994: 205, 1998: 3; Dalla Montà et al. 1993; Coscollá 1997: 214; Colombera et al. 2001: 94; Marchesini et al. 2006: 12; Bagnoli and Lucchi 2006: 140; Marchesini 2007: 41; Lucchi and Santini 2011: 199; Loni et al. 2016: 131; Scaramozzino et al. 2017b: 132.
Campoplex difformis : Thompson 1946: 484.
Omorgus difformis Gmelin: Catoni 1910: 17, 1914: 250; Silvestri 1912: 295; Schwangart 1913: 6, 1918: 547; Ruschka and Fulmek 1915: 391; Leonardi 1925: 259; Boselli 1928: 189; Stellwaag 1928: 663.

######## Italian distribution of reared parasitoids.

Trentino-South Tyrol: Catoni 1910, 1914; Silvestri 1912; Schwangart 1913, 1918; Ruschka and Fulmek 1915.

Veneto: Marchesini and Dalla Montà 1992, 1994, 1998; Dalla Montà et al. 1993; Marchesini et al. 2006; Marchesini 2007.

Piedmont: Colombera et al. 2001.

Tuscany: Dalla Montà et al. 1993; Bagnoli and Lucchi 2006; Lucchi and Santini 2011; Loni et al. 2016; Scaramozzino et al. 2017b.

Campania: Silvestri 1912.

Sicily: Alcamo (TP), ex *Lobesia botrana* on grapes (ZSM new record).

######## Distribution.

*Campoplex capitator* is a Mediterranean species, occurring in the Iberian Peninsula, France, Corsica, Italy, Switzerland and Turkey (Yu et al. 2012; Zwakhals and van Achterberg 2017). It is widespread in most of the southern European wine-growing areas (Bagnoli and Lucchi 2006), although its presence on EGVM in Southern Italy was not definitely ascertained. Nuzzaci and Triggiani (1982), in Apulia, underline the presence of *C. difformis* on EGVM feeding on *Daphne gnidium* and the absence of *C. capitator*, as already stated by Silvestri (1912). When checking his collection in Naples, we found two series of specimens, both reported as *C. difformis* from *L. botrana*. Actually, the two series are composed of at least three different species:

1. *Campoplex capitator* from Portici (Naples), 5 females and 3 males, and from San Michele all’Adige (Trento), 3 females, 3 males and 1 individual without metasoma. The specimens from San Michele all’Adige have the same origin of those studied and published by Catoni (1910) with the name of *C. difformis* and identified by O. von Schmiedeknecht.

2. *Diadegma stigmatellae* Horstmann, 1980 (Campopleginae), 6 males and 4 females from Portici, a parasitoid of Gracillariidae (Shaw and Horstmann 1997).

3. *Pristomerus vulnerator* (Panzer, 1799) (Cremastinae) 1 male and 1 female from Portici.

We are not sure if the two series of specimens correspond to those actually studied by Silvestri but we think that the *Campoplex* specimens he had attributed to *Omorgus difformis* belong to *C. capitator*.

In the Horstmann collection, as well as in the general collection of ZSM, we found 7 females and 6 males of *C. capitator* from Sicily (Alcamo, TP), emerged from larvae of *L. botrana* feeding on grapes in July 2007, August 2009 and late May-June 2010. Also in the Horstmann collection we examined a male and a female of *C. capitator* from Piacenza (Northern Italy), obtained from *E. ambiguella*.

######## Host range.

*Campoplex capitator* seems to have an extremely limited host range. It was discovered on EGVM for the first time by Coscollá (1980) in Spain. Yu et al. (2012) list only two host species, *L. botrana* and *Ancylis mitterbacheriana* (Denis & Schiffermüller, 1775) (Lepidoptera Tortricidae). According to Villemant et al. (2011), in French vineyards *C. capitator* lives mainly at the expenses of *L. botrana* and *E. ambiguella*, though it has been obtained occasionally also from *S. pilleriana*. All the mentioned hosts live mainly on the grapevine, with the exception of *A. mitterbacheriana*, an univoltine leaf folder which lives on deciduous woodlands and whose larvae feed on the leaves of beech, common hornbeam, oaks, and sweet chestnut (Alford 2012, Brown et al. 2008).

######## Ecological role.

**Figure 4.**
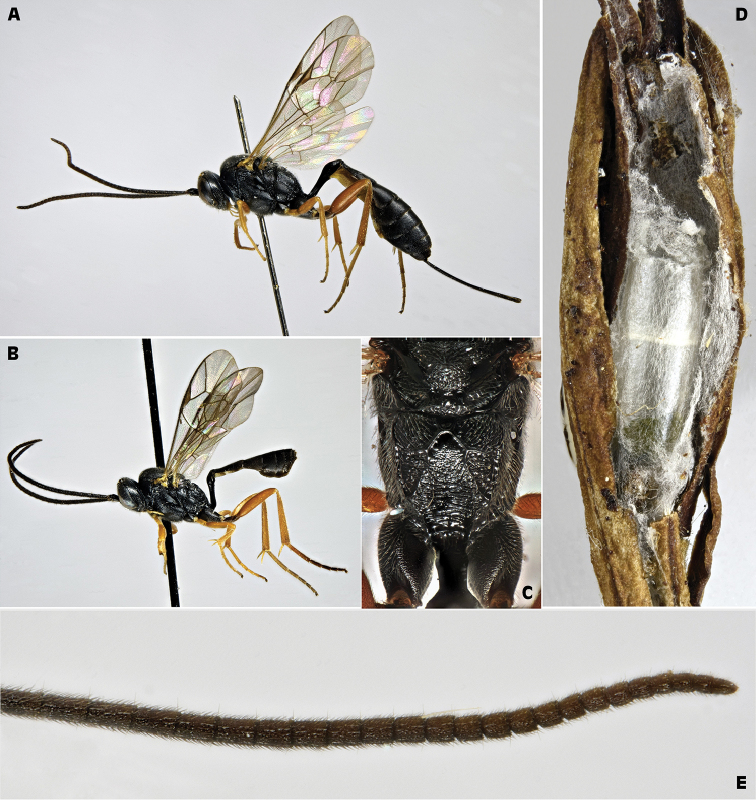
*Campoplex capitator* Aubert, 1960 (female and male: IT, San Rossore Pisa ex *Lobesia botrana*) **A** female habitus lateral view **B** male habitus lateral view **C** propodeum female, dorsal view **D** opened cocoon inside a EGVM cocoon, in a dried nest on *Daphne gnidium* **E** distal 24 articles of female antenna.

*Campoplex capitator* is a solitary koinobiont larval endoparasitoid. Its development is strongly synchronized with *L. botrana*: both species overwinter in the same places, and live in close association, the first at the expense of the larvae of all the moth generations. The female oviposits into the body of EGVM larvae of 2nd-4th instar (Thiéry 2008, Villemant et al. 2011). Endophagous larva kills the host after spinning its own cocoon inside the moth cocoon. The larva of *C. capitator* builds a delicate elongated semi-transparent cocoon characterized by rounded poles, white color and a thin median opaque transverse line (Figure 4D).

The parasitization rates recorded in Europe (Italy excluded) are shown in Table 5, while those recorded in Italy are shown in Table 6.

**Table 5.** *Campoplex capitator* parasitization rates recorded on the European grapevine moth in Europe (Italy excluded).

Author/s and publication year	Country and region	Host plant or cv	Year	1^st^ generation (antophagous)	2^nd^ generation (carpophagous)	3^rd^ generation (carpophagous)
Carlos et al. 2006	Portugal, Douro, S. Luiz	grapevine	2002	6.98%	3.88	–
Carlos et al. 2006	Portugal, Douro, Bonfim	grapevine	2002	5.5%	–	–
Coscollá 1980	“comarcas vitícolas valencianas”*	grapevine	1978	4.62%	–	–
Coscollá 1980	“comarcas vitícolas valencianas”*	grapevine	1979	1.35%	<0.75%	0.79%
Coscollá 1980	“comarcas vitícolas valencianas”*	grapevine	1980	1%	0	0
Moreau et al. 2010	Switzerland, Yvorne (VD)	Grapevine cv Chasselas	2003	1.53%	–	–
Moreau et al. 2010	Switzerland, Yvorne (VD)	Cv Pinot noir	2003	3.63%	–	–
Moreau et al. 2010	Switzerland, St Pierre-de-Clages (VS)	Chasselas	2003	1.12%	–	–
Moreau et al. 2010	France, Tavel (SF)	Grenache	2003	17.6%	–	–
Moreau et al. 2010	France, Roquemaure (SF)	Syrah	2004	0.56%	–	–

* average figure on three vineyards, recalculated

**Table 6.** *Campoplex* species: percentages of parasitism on the European grapevine moth reported in Italy by different authors.

Species or Author/s and publication year	Italian Region/ Locality	Host plant	Year	1^st^ generation (antophagous)	2^nd^ generation (carpophagous)	3^rd^ generation (carpophagous)
***Campoplex* sp.**						
Pinna et al. 1989	Piedmont/ Ghemme (NO)	grapevine	1986/87	–	2.46*	does not occur
Pinna et al. 1989	Piedmont/ Piverone and Caluso (TO)	grapevine	1985/86	–	1.78*	does not occur
Pinna et al. 1989	Piedmont/ Piverone and Caluso (TO)	grapevine	1986/87	–	13.28*	does not occur
Pinna et al. 1989	Piedmont/ Riccaldone e Castelrocchero (AL)	grapevine	1986/87	–	18.24*	does not occur
Pinna et al. 1989	Piedmont/ Mango e Castellinaldo (CN)	grapevine	1986/87	–	6.33*	does not occur
Lozzia and Rigamonti 1991	Piedmont/ Ghemme (NO)	grapevine	1988	–	12.50	does not occur
***Campoplex capitator***						
Colombera et al. 2001	Piedmont/ Caravino (IPM)	grapevine	1998	7.4*	0	does not occur
Colombera et al. 2001	Piedmont/ Caravino (IPM)	grapevine	1999	3.07*	1.9*	does not occur
Colombera et al. 2001	Piedmont/ Settimo Vittone (Convent.)	grapevine	1998	0.61*	0	does not occur
Colombera et al. 2001	Piedmont/ Settimo Vittone (Convent.)	grapevine	1999	1.85*	5.88*	does not occur
Marchesini and Dalla Montà 1994	Veneto/ Pernumia (PD)	grapevine	1988	–	–	1.46
Marchesini and Dalla Montà 1994	Veneto/ Pernumia (PD)	grapevine	1989	12.17	3.15	9.4
Marchesini and Dalla Montà 1994	Veneto/ Pernumia (PD)	grapevine	1990	11.78	0.46	0
Marchesini and Dalla Montà 1994	Veneto/ Pernumia (PD)	grapevine	1992	0	0	0.02
Marchesini and Dalla Montà 1994	Veneto/ Colognola (VR)	grapevine	1990	0.74	0.74	2.69
Marchesini and Dalla Montà 1994	Veneto/ Colognola (VR)	grapevine	1991	0.33	0	3.52
Marchesini 2006 and 2007	Veneto	grapevine	2000 (2)		3.8/14.3	6.4/8.3
Marchesini 2006 and 2007	Veneto	grapevine	2001 (2)	0/6.0	13.0/10.0	0.5/2.0
***Campoplex difformis***						
Nuzzaci and Triggiani 1982	Apulia/ Monopoli (BA) and Martinafranca (TA)	*Daphne gnidium*	1979–82		4**	

* recalculations on the basis of data provided by the authors.** highest value recorded in three years of observations.

Silvestri (1912) frequently found *C. capitator* on EGVM, both in Trentino (Northern Italy), in spring, and in Portici (Naples), from July to September. In Veneto (Northern Italy) it attacks all the generations of EGVM, with irregular and not particularly high rate of parasitism, often less than 1%, sometimes close to 12% in the first generation and 14% in the second and slightly more than 8% in the third generation (Marchesini and Dalla Montà 1994, Marchesini et al. 2006); sometimes it is absent. In Piedmont, where EGVM developed 2 generations per year, Colombera et al. (2001) recorded parasitization rates of 7.4% and 5.9%, respectively. In Tuscany (Central Italy), on grapevine, *C. capitator* is the most frequent species among larval parasitoids, showing a good parasitic activity throughout the region, mostly on larvae of the first two generations of the year (Bagnoli and Lucchi 2006). In the Natural Reserve of San Rossore (Pisa, Tuscany), it is very frequent on *Daphne gnidium*, where it represents the dominant species in the parasitoid community of EGVM; attacking larvae of all three generations, it contributed for more than 58% of the total number of parasitoids found in 2014 and more than 73% in 2015, with an overall annual parasitization rate for 2014 next to 10% (Loni et al. 2016).

In France, the rates of parasitism can be very high, especially in the EGVM first generation (Villemant et al. 2011). In the vineyards of Valencia (Spain), *C. capitator* is the only larval parasitoid that plays a significant role in the control of EGVM, even if the total parasitism levels found in that region were low across all the three generations (Coscollá 1997). In Douro Wine Region (Portugal), *C. capitator* is the second most abundant parasitoid of EGVM (Carlos et al. 2013), representing the 11.8% of parasitoids obtained in 10-year surveys. In Turkey, the species is mostly widespread in the Aegean vineyards (Koclu et al. 2005, Özsemerci et al. 2016).

Despite being considered one of the possible candidates for use in the biological control of EGVM, the knowledge about its behavior and its development are too limited and still some difficulties have to be overcome to develop an efficient mass rearing in bio-factory (Bagnoli and Lucchi 2006). Nevertheless, a recent cooperation between Italian and Chilean entomologists seems very promising (Lucchi et al. 2017).

######## Taxonomic notes.

Horstmann (1985) divided the Western Palaearctic species of the genus *Campoplex* in four species-groups: *melanostictus* (including the *spurius*-group), *continuus*, *discrepans* and *difformis* species-groups; later, the *C. borealis* species-group was added (Horstmann 2012). Both *Campoplex capitator* and *C. difformis* belong to “*difformis*” species-group, which is characterized by occipital carina joining hypostomal carina at a right angle at the base of the mandibles (i.e., occipital carina turned outwards ventrally); slender body with apically compressed metasoma; hind tibia with the median outer part from yellowish red to reddish brown, and basal part not clearly brightened; ovipositor sheath relatively long (at least as long as the hind tibia). Horstmann (1985) describes the female of *C. capitator* as follows in the key: body size approx. 5 mm, face wider than long, temples behind the eyes narrow, the lines (as seen in profile from above) touching the outside of the eyes and temples usually intersecting in or behind the scuto-scutellar groove, antennal segments, in the last quarter, at least as long as wide, prepectal carina medially not significantly broader than ventrolaterally and not clearly notched, hind coxae black with hind femora predominantly red, ovipositor sheath 1.4–1.7 times as long as the hind tibia, second metasomal segment not more than 1.6 times as long as wide.

The females of *C. difformis*, which are very similar to those of *C. capitator*, in Horstmann’s keys are distinguished by: body size approx. 8 mm, face longer than wide, antennal segments, in the last quarter, much wider than long, and the area superomedia of propodeum wide, and not clearly separated from the area petiolaris; both areas are clearly depressed.

Villemant et al. (2011) pointed out that *C. capitator* has often been confused with *C. difformis* in the past and many reports of this species on EGVM should probably be related to *C. capitator*. The identifications of *C. difformis* made by Silvestri as well as those of Catoni are to be referred to *C. capitator* (see above). The record of Nuzzaci and Triggiani (see *C. difformis*), whose specimens were identified by Horstmann, has to be considered correct.

Molecular-based studies indicate that *C. capitator* could be conspecific of *C. formosanae* Horstmann, 2012, a species reared on *Enarmonia formosana* in Germany (Hunt and Kuhlmann 2007, Hunt et al. 2008). The species was identified as *C. dubitator* at first, but then recognized as a valid species by Horstmann (2012). Despite molecular differences between the two species were not significant (Hunt and Kuhlmann 2007, Hunt et al. 2008), laboratory tests showed that *C. formosanae* was unable to develop on EGVM larvae and small but constant morphological characters have been found that lead to consider *C. formosanae* as a distinct species from *C. capitator* (Hunt et al. 2008, Jenner et al. 2013).

**Figure 5.**
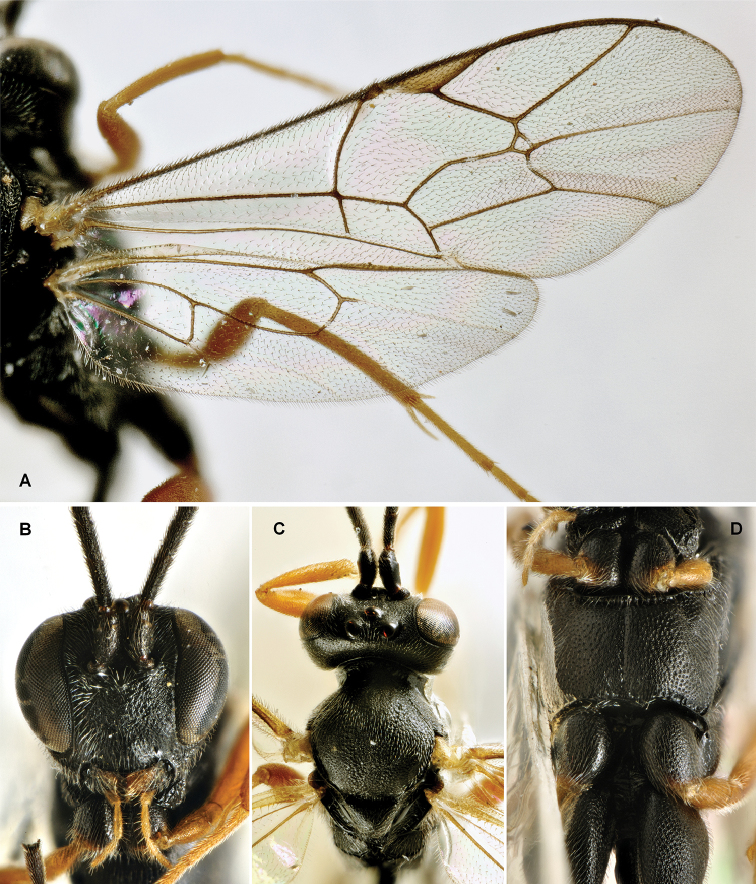
*Campoplex capitator* Aubert, 1960 (female: IT, San Rossore Pisa ex *Lobesia botrana*) A wings B head frontal view C head and mesosoma dorsal view D mesopleuron with epicnemial and postpectal carinae, ventral view.

####### Campoplex difformis

Taxon classificationAnimaliaHymenopteraIchneumonidae

(Gmelin, 1790)

Campoplex difformis : Nuzzaci and Triggiani 1982: 49.

######## Italian distribution of reared parasitoids.

Apulia: Nuzzaci and Triggiani 1982 [on *Daphne gnidium* L.].

######## Distribution.

The species is present throughout Europe up to the Caucasus and Uzbekistan, the Canary Islands and Madeira, Tunisia and Greenland (Yu et al. 2012; Zwakhals and van Achterberg 2017).

######## Host range.

Yu et al. (2012) list 64 host species belonging to 18 different families (15 of Lepidoptera and 3 of Hymenoptera). This long list has to be verified, because in the past the specific interpretation of *C. difformis* was rather uncertain (see taxonomic notes under *C. capitator* and Horstmann 1985). The most represented family is that of Tortricidae, with 35 species (including *L. botrana* and *E. ambiguella*). Tortricids could be actually the only hosts of *C. difformis*, because all known hosts of the “*difformis*” species-group belong to this family (Horstmann 1985). *Archips podana* (Scopoli, 1763) was the only host ascertained for this species in the work of Horstmann (1985). In Evenhuis and Vlug (1983), a hypothetical *Campoplex difformis*, so identified by Horstmann, is reported attacking three other tortricid species, *Pandemis cerasana* (Hübner, 1786), *Adoxophyes orana* (Fischer v. Röslerstamm, 1834) and *Acleris rhombana* (Denis & Schiffermüller, 1775).

######## Ecological role.

*Campoplex difformis* is a koinobiont larval endoparasitoid often reported as a parasitoid of *E. ambiguella* in France (Voukassovitch 1924). Its larva kills the moth larva when it is ready to pupate, and weaves its own cocoon next to the host spoils (Marchal 1912, Voukassovitch 1924). In Apulia (Southern Italy), this species occurred frequently, showing a parasitism rate of approx. 4% on EGVM larvae feeding on *Daphne gnidium* (Nuzzaci and Triggiani 1982). The species has been reported on *L. botrana* in Austria, France, Germany, Russia, Spain and Bulgaria, as well as in Italy (Thompson 1957, Zapryanov and Stoeva 1982, Zapryanov 1985).

*Campoplex difformis* is itself the victim of *Dibrachys microgastri* (Bouché, 1834) (Hymenoptera Pteromalidae) (Faure and Zolotarewsky 1925, Zapryanov and Stoeva 1982), *Perilampus tristis* Mayr, 1905 (Hymenoptera Perilampidae) (Thompson 1958) and *Cirrospilus* sp. (Hymenoptera Eulophidae) (Noyes 2017).

######## Taxonomic notes.

*Campoplex difformis* (Gmelin) was designated as the type species of the genus *Campoplex* Gravenhorst, 1829 by Westwood (1840). It also gives the name to a complicated group of very similar species, with morphological characteristics insufficient to allow a definitive identification (Jenner et al. 2013). In the past this species was mainly attributed to the genera *Limneria* Holmgren, 1859 and *Omorgus* Förster, 1869 (= *Omorga* Thomson, 1887). Unfortunately, the interpretation of the species *Ichneumon difformis* Gmelin, until the studies of K. Horstmann (1969, 1985), has been uncertain. The type of Gmelin was destroyed. Then, following the first interpretation of the species given by Gravenhorst (1829), Horstmann fixed the lectotypus of *Limneria mutabilis* Holmgren, in Holmgren’s collection in Stockholm as a neotypus of *C. difformis* (Horstmann 1969, 1985). Thus, *C. mutabilis* of Holmgren became a junior synonym of *C. difformis*, and *C. difformis* *sensu* Holmgren (and Thomson) became *Campoplex deficiens* Gravenhorst, 1829. Therefore, the interpretation of the species given by Gravenhorst (1829) [and hence by Horstmann (1985)] differed from that of other taxonomists (mainly Holmgren, Thomson and Schmiedeknecht), who considered *C. difformis* and *C. mutabilis* two distinct species. For this reason Aubert (1971, 1974 and 1981), another leading authority in the ichneumonid taxonomy, rejected the neotypus fixed by Horstmann and created another typus in the collection of Thomson in Lund, in order to keep *C. mutabilis* as a separate species from *C. difformis*. Consequently, *C. deficiens* Gravenhorst became synonym of *C. difformis* (see Table 7). In this work we follow the interpretation of Gravenhorst (1829), Horstmann (1969, 1985), and Yu and Horstmann (1997). *Campoplex difformis* has three synonyms: *Campoplex lineolatus* Ratzeburg, 1844, *Limneria mutabilis* Holmgren, 1860 and *Nepiera algerica* Habermehl, 1922, and a variety with dark hind legs (var. *obscuripes* Greese, 1927).

**Table 7.** Different interpretations and synonyms attributed by Horstmann (1969) and Aubert (1971) to the triplet *C. difformis*, *C. mutabilis*, and *C. deficiens*.

Species, named as in the original descriptions	Interpretation given by Horstmann (1969), following Gravenhorst	Interpretation given by Aubert, following Holmgren, Thomson, and Schmiedeknecht
*Ichneumon difformis* Gmelin, 1790	*Campoplex difformis*, species valida	*Campoplex difformis*, species valida
*Limneria mutabilis* Holmgren, 1860	Junior synonym of *Campoplex difformis*	*Campoplex mutabilis*, species valida
*Campoplex deficiens* Gravenhorst, 1829	*Campoplex deficiens*, species valida	Junior synonym of *Campoplex difformis*

####### Diadegma armillata

Taxon classificationAnimaliaHymenopteraIchneumonidae

(Gravenhorst, 1829)

Figure 6

Angitia tibialis : Silvestri 1912: 296; Leonardi 1925: 259.
Angitia (Dioctes) tibialis : Stellwaag 1928: 664.

######## Italian distribution of reared parasitoids.

Campania: Silvestri 1912.

######## Distribution.

*Diadegma armillata* is a Palaearctic widespread species. It is found throughout Europe, Middle East, Caucasus, Kazakhstan, China and Korea (Yu et al. 2012; Zwakhals and van Achterberg 2017).

######## Host range.

It is an important koinobiont larval endoparasitoid of macro- and microlepidoptera (Arctiidae, Argyresthiidae, Choreutidae, Coleophoridae, Gelechiidae, Geometridae, Gracillariidae, Lymantriidae, Noctuidae, Pieridae, Plutellidae, Psychidae, Pterophoridae, Pyralidae, Simaethidae, Tortricidae, Yponomeutidae). Yu et al. (2012) list 57 host species. Further three species have to be added to the list: *Celastrina argiolus* (Linnaeus, 1758) (Lycaenidae), *Acrobasis marmorea* (Haworth, 1811) (Pyralidae) and *Swammerdamia caesiella* (Hübner, 1796) (Yponomeutidae) (Shaw et al. 2016).

*Diadegma armillata* is particularly active against various *Yponomeuta* spp., attacking crop fruits, and it was introduced in 1989–1991 from France to northwestern Washington (USA), to control the apple ermine moth, *Yponomeuta malinellus* (Zeller, 1838) without becoming established (Unruh et al. 2003).

######## Ecological role.

*Diadegma armillata* is a multivoltine species. Silvestri (1912) obtained few specimens of this wasp from EGVM cocoons in Portici (Naples). The record of Silvestri remains the only one for this species on EGVM.

######## Taxonomic notes.

The first serious attempt to bring order in the existing confusion for the interpretation of the European species of the genus *Diadegma* Förster, 1869 is due to the efforts of Horstmann (1969), who revised many types of the described species. The identifications of most species were based on poor morphological characters and are unfortunately unreliable (Horstmann 1969).

*Diadegma armillata* belongs to the subgenus Nythobia Förster, 1869, which includes group species with the seventh metasomal tergite deeply notched medially, the ovipositor sheath longer than the first metasomal tergite and shorter than the hind tibia (Horstmann 1969). The female of *D. armillata* is distinguished from the related species by a head with strongly narrowed temples; last article of the antennae longer than wide; propodeum with area superomedia wider than long and opened posteriorly, costulae strong; petiolar area slightly sunken and transversely striated; first metasomal segment with slightly protruding spiracles and postpetiole with parallel sides; ovipositor sheath approx. twice the length of the first metasomal segment; front and middle coxae yellow, the middle ones sometimes darkened at the base; femora and tibiae reddish yellow; hind tibia externally dark brown with white-yellow spots at the base and in the middle; metasoma variably stained with red (Horstmann 1969).

Some doubts regarding the distribution and host range of *D. armillata* arises from the fact that *D. semiclausum* (Hellen, 1949), a common parasitoid of the diamondback moth *Plutella xylostella* (Linnaeus, 1758), has been misidentified with *D. tibialis* (Gravenhorst, 1829), which is currently a synonym of *D. armillata* (Horstmann 1969, Azidah et al. 2000). Under the name *tibialis*, *D. semiclausum* was introduced in 1951 from Italy to Australia to control the diamondback moth (Oatman 1978).

**Figure 6.**
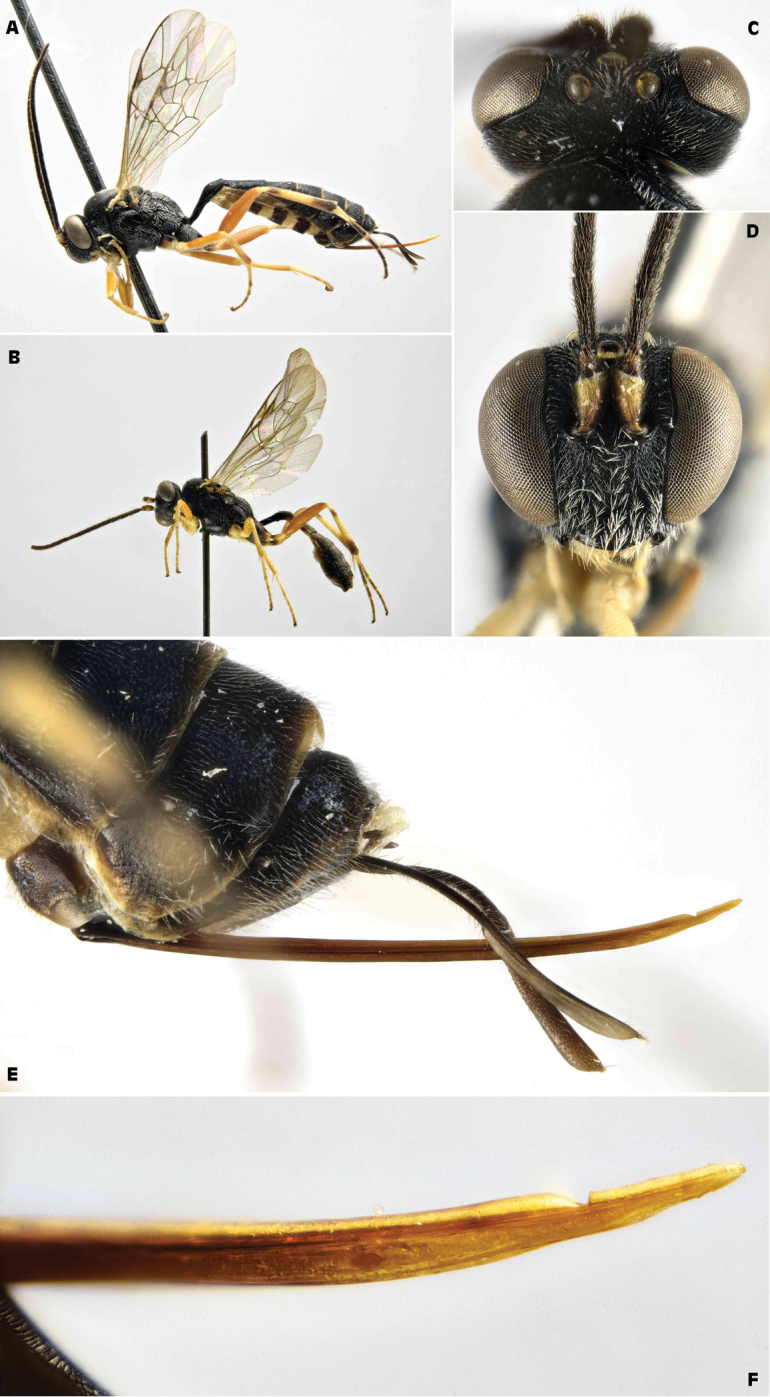
*Diadegma armillata* (Gravenhorst, 1829) (female: D, Ostfriesischen Inseln, Mollum Memmert, July 24–30, 1986, leg. V. Haeseler; male: IT, South Tyrol, Kaltern, July 29, 1959; ZSM) **A** female habitus lateral view **B** male habitus lateral view **C** female head dorsal view **D** female head frontal view **E** female tergites 7–9 of metasoma with ovipositor and ovipositor sheath, lateral view **F** female ovipositor with subapical dorsal notch lateral view.

####### Diadegma tenuipes

Taxon classificationAnimaliaHymenopteraIchneumonidae

(Thomson, 1887)

Angitia tenuipes : Catoni 1910: 17, 1914: 250; Schwangart 1913: 6; Boselli 1928: 189; Stellwaag 1928: 664; Thompson 1946: 483.

######## Italian distribution of reared parasitoids.

Trentino-South Tyrol: Catoni 1910, 1914; Schwangart 1913.

######## Distribution.

A Palaearctic species ranging from Europe up to Mongolia; in Europe it is widely present with the exception of the Iberian Peninsula, Balkans and Greece. In North Africa it is reported only from Tunisia (Yu et al. 2012; Zwakhals and van Achterberg 2017).

######## Host range.

*Diadegma tenuipes* is a solitary koinobiont larval endoparasitoid of a dozen of hosts, which belong to the Lepidoptera families Coleophoridae, Momphidae, Pieridae, Plutellidae, Psychidae, Tortricidae and to the Hymenoptera families Tenthredinidae and Braconidae (Yu et al. 2012). Among these, some hosts of economic importance are indicated as the diamondback moth, the Oriental fruit moth (*G. molesta*), the European grape berry moth (*E. ambiguella*) and EGVM.

######## Ecological role.

The only Italian records of *D. tenuipes* on EGVM are those of Catoni (1910 and 1914). In the vineyards of southern Romania, Bărbuceanu and Jenser (2009) found this species, along with three other species of the same genus, attacking the overwintering generation of EGVM, with a rather low parasitization rates (0.8%). In Romania it is reported by Petcu (1978) living on *E. ambiguella* as well.

######## Taxonomic notes.

Like the previous species, *D. tenuipes* has been assigned by Horstmann (1969) to the subgenus Nythobia. It measures approx. 6 mm in length, with the head posteriorly narrowed, propodeum with evident costulae (anterior transverse carinae), and the area superomedia shorter than twice its width; areolet of the fore wing rather large and intercepted by the second recurrent vein (2m-cu) after the middle; mesopleuron with speculum almost smooth, very shiny, the area close to mesopleural suture finely dotted; the seventh metasomal tergite dorsally deeply notched, ovipositor sheath 0.8 times the length of the hind tibia and 1.4 times that of the first metasomal tergite; body black, wings with pterostigma light brown, fore coxae light, femora and tibiae reddish yellow, posterior tibiae dark behind the base and at the apex, sides of the third metasomal tergite stained with red (Horstmann 1969).

####### Nemeritis

Taxon classificationAnimaliaHymenopteraIchneumonidae

ssp. Holmgren, 1860

Nemeritis sp.: Del Guercio 1899: 155–156.

######## Italian distribution of reared parasitoids.

Tuscany: Del Guercio 1899.

######## Taxonomic notes.

The limited information provided by Del Guercio (1899) does not allow designation of the two species of *Nemeritis* to any of the parasitoids associated to EGVM. Though Leonardi (1925) included the work of Del Guercio in his bibliography, he did not quote these species, which are not mentioned by any other author.

The species of *Nemeritis* have been divided by Horstmann (1975) in four groups: *caudatula*- and *elegans*-group, which parasitizes Raphidioptera, *macrocentra*-group which parasitizes Coleoptera (Cleridae, Malachiidae) and Lepidoptera and *lissonotoides*-group for which no host records are available (Horstmann 1994). Even though most of the species seem to attack concealed hosts under the bark or in bark crevices (Horstmann 1975), few species of the *macrocentra*-group have been recorded on moth species of economic importance, like the Mediterranean flour moth (*Ephestia kuehniella* Zeller, 1879), the European grain moth (*Nemapogon granella* (Linnaeus, 1758)) or the strawberry fruitworm (*Cnephasia longana* (Haworth, 1811)) (Horstmann 1994; Yu et al. 2012).

In the past, the genus *Nemeritis* included species of other campoplegine genera like *Campoplex*, *Cymodusa* or *Venturia* (Thomson 1887, Schmiedeknecht 1909). It is possible that the two species cited as *Nemeritis* sp. by Del Guercio (1899) are actually *Venturia canescens* (Gravenhorst, 1829). Schmiedeknecht, who identified ichneumonids obtained by Del Guercio (1899, page 156), refers to *Venturia canescens* as *Nemeritis canescens* in his fundamental work on European ichneumonids (Schmiedeknecht 1909, page 1688). At least the general habitus and wing venation in the picture of the second species (Del Guercio 1899, page 156) fit with the general aspect of *V. canescens*.

####### Sinophorus crassifemur

Taxon classificationAnimaliaHymenopteraIchneumonidae

(Thomson, 1887)

Eulimneria crassifemur : Schwangart 1918: 547, Stellwaag 1928: 663.
Eulimneria ramifemur : Schwangart 1913: 6.

######## Italian distribution of reared parasitoids.

Trentino-South Tyrol: Schwangart 1913, 1918; Stellwaag 1928.

######## Distribution.

Transpalaeartic species widespread in Europe, present in Caucasus and Central and Far Eastern Russia as well (Yu et al. 2012; Zwakhals and van Achterberg 2017). The report for India by Morley (1913) needs to be confirmed.

######## Host range.

Yu et al. (2012) list 14 host species for *S. crassifemur*, including 2 Geometridae, 2 Pieridae, 3 Pyralidae, 3 Tortricidae (Lepidoptera) and 4 species of Pamphiliidae (Hymenoptera).

######## Ecological role.

The very little information available on this species derives from Stellwaag (1928), who reports that it has been obtained by Schwangart and Catoni in April. Nevertheless, Catoni (1910, 1914, 1915), Leonardi (1925) and Boselli (1928) do not mention this species in their lists. Thompson (1946) quotes it in Austria based on an article of Schwangart (1918) who obtained it from EGVM and *E. ambiguella* in Trentino. Hoffmann and Michl (2003) do not cite it. The species was also obtained from EGVM in Bulgaria (Athanassov 1981, Zapryanov 1985).

######## Taxonomic notes.

Very likely the species referred as *Eulimneria crassifemur* Thomson by Schwangart (1918) and Stellwaag (1928) is *Sinophorus turionum* (Ratzeburg, 1844). Carlson (1979) writes: “…in literature published before the description of *alkae* in 1928 [*Sinophorus alkae* (Ellinger and Sachtleben, 1928) is a junior synonym of *S. turionum*] and in some literature for more than ten years thereafter, the species was misidentified as *crassifemur* (Thomson)”. Sanborne (1984), in his review of the world species of the genus *Sinophorus* Förster, 1869, indicates among the hosts of *S. crassifemur*, only the web-spinning larvae of *Cephalcia* sp. and *Acantholyda* sp. (Hymenoptera Pamphiliidae) on *Pinus* spp.

####### Sinophorus turionum

Taxon classificationAnimaliaHymenopteraIchneumonidae

(Ratzeburg, 1844)

Figure 7

Eulimneria alkae : Thompson 1946: 484.

######## Italian distribution of reared parasitoids.

The indication of this species on EGVM is due to Thompson (1946) that found it in a compendium of Hymenoptera parasitoids of European corn borer of Chu and Hsia (1937) that, unfortunately, we were not able to examine. Thompson and Parker (1928), on the basis of a record by Paillot (1924), report this species under the name of *Eulimneria crassifemur* Thomson, both on *L. botrana* and on *E. ambiguella*.

######## Distribution.

The species is widely distributed throughout the Palearctic region, except North Africa (Yu et al. 2012). It has been introduced several times in the United States and Canada for the biological control of the European corn borer *O. nubilalis* (Bartlett et al. 1978, Carlson 1979) and the pine shoot borer, *R. buoliana* (Syme 1971, 1984, Carlson 1979) without being established (Sanborne 1984). Its presence in the Oriental region (India and Sri Lanka) (Townes et al. 1965, Yu and Horstmann 1997) has to be confirmed.

######## Host range.

Yu et al. (2012) list 26 host species, belonging to nine families of Lepidoptera and one family of Hymenoptera. Tortricidae and Pyraloidea are the most represented. Five other lepidopteran host species need to be added: *Anania hortulata* (Linnaeus, 1758), *Nascia cilialis* (Hübner, 1796), *Pyrausta aurata* (Scopoli, 1763) (Crambidae), *Gymnoscelis rufifasciata* (Haworth, 1809) (Geometridae) and *Acrobasis advenella* (Zincken, 1818) (Pyralidae) (Shaw et al. 2016).

######## Ecological role.

It is reported as one of the main parasitoids of the European corn borer in Europe (Thompson and Parker 1928) under the name *Eulimneria crassifemur*, and in the Northern part of Far East (Manchuria and North Korea) (Clark 1934) as *Eulimneria alkae*. In the case of *L. botrana*, it is certainly an occasional parasitoid of minor importance, perhaps a secondary adaptation to a host different from the usual ones. In the literature we found, besides those mentioned for Italy, scattered reports of his presence on EGVM, mostly under the name *crassifemur* in its various generic combinations: in Austria (Thompson and Parker 1930), France (Paillot 1924, Thompson and Parker 1928), Bulgaria (Athanassov 1981, Zapryanov 1985), Germany (Thompson 1946) and Spain (Thompson 1946, Coscollá 1997). Another species, *S. costalis* (Thomson, 1887), has been recorded on EGVM in Moldavian vineyards, Romania (Pisicá and Páişescu-Bărbuceanu 2002, Bărbuceanu and Jenser 2009).

######## Taxonomic notes.

Many authors dealt with this species, especially in relation to its main hosts, *O. nubilalis* and *R. buoliana*. The species has been often confused with *Limnerium crassifemur* (recte *Sinophorus crassifemur*) (Paillot 1924, Thompson and Parker 1928, Goidanich 1931, Carlson 1979), which, instead, is mainly related to species of the family Pamphiliidae (Hymenoptera) (Sanborne 1984; see notes on previous species). The name *crassifemur* was later corrected in *alkae* by Ellinger and Sachtleben (1928), which then turned out to be a synonym of *Sinophorus turionum* (Sanborne 1984). It is likely that the report of Thompson (1946) for Italy on EGVM, quoted by Schwangart (1918) and Stellwaag (1928), concerns the previous species (see above).

**Figure 7.**
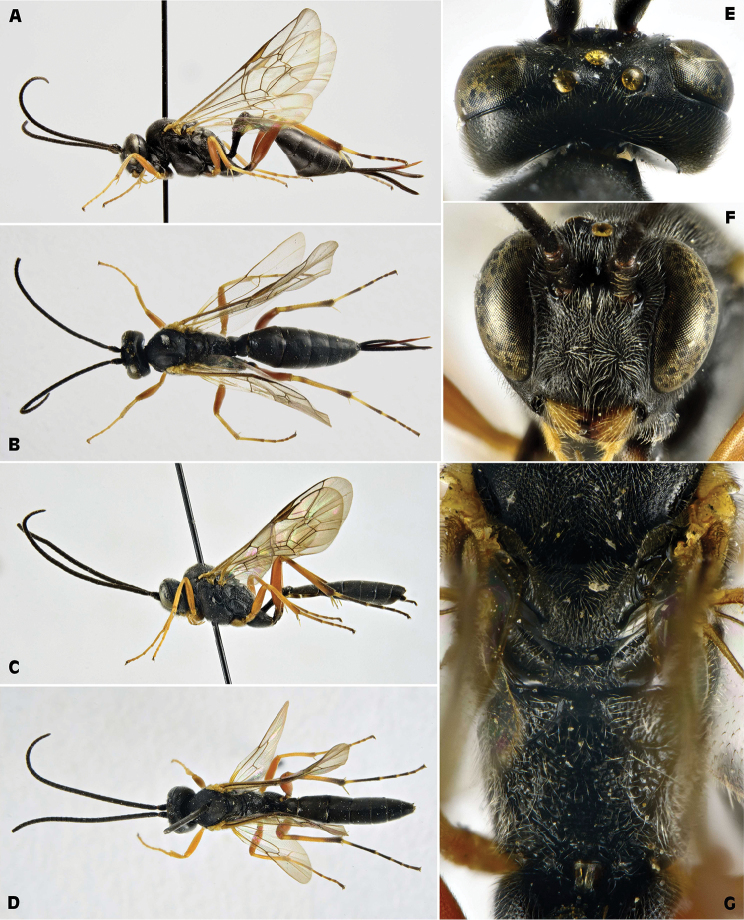
*Sinophorus turionum* (Ratzeburg, 1844) (female and male: IT, Lavariano, Udine, August 29, 1983 and September 26, 1983, ex *Ostrinia nubilalis*, ZSM) **A, B** female habitus lateral and dorsal view, respectively **C, D** male habitus lateral and dorsal view, respectively **E** female head dorsal view **F** female head frontal view **G** female propodeum dorsal view.

####### Tranosemella praerogator

Taxon classificationAnimaliaHymenopteraIchneumonidae

(Linnaeus, 1758)

Figure 8

Tranosemella praerogator : Dalla Montà et al. 1993; Marchesini and Dalla Montà 1994: 205, 1998: 3; Coscollá 1997: 215; Colombera et al. 2001: 94; Marchesini et al. 2006: 12; Marchesini 2007: 41, 48.

######## Italian distribution of reared parasitoids.

Veneto: Dalla Montà et al. 1993; Marchesini and Dalla Montà 1994, 1998; Marchesini et al. 2006; Marchesini 2007.

Piedmont: Colombera et al. 2001.

######## Distribution.

Holarctic widespread species (Iceland, Europe, Egypt, Central Russia and the Far East, Japan, Canada, United States) (Yu et al. 2012). Its presence in India (Morley 1913) has to be confirmed, since it is most probably based on a misidentification for a Tryphoninae Ichneumonid species belonging to the genus *Dyspetes* Förster, 1869 (see taxonomic notes).

######## Host range.

Yu et al. (2012) list 44 species of Lepidoptera hosts, especially Tortricidae (32 species), including various species of the genera *Archips*, *Argyrotaenia*, *Choristoneura* and *Pandemis*. The list reports also Plutellidae, including *P. xylostella*, Gelechiidae as *Pectinophora gossypiella* (Saunders, 1844), Yponomeutidae, Sesiidae, Pyralidae, Arctiidae, Geometridae and Noctuidae.

######## Ecological role.

In Italy, it has been reported on grapevine in Veneto and Piedmont. In Veneto (Marchesini and Dalla Montà 1994) it was regularly obtained from EGVM larvae of first and second generation, with rates of parasitism higher than those of *C. capitator* (Table 8). It has never been collected in the EGVM third generation, and it is supposed to overwinter on alternative hosts. In contrast, Colombera et al. (2001) found it in the first generation, with low levels of parasitization (lower than 1%), while *C. capitator* showed a more important and incisive activity. In Veneto, the species was hyperparasitized by *Elasmus steffani* Viggiani, 1967 (Hymenoptera Elasmidae), in turn attacked by *Baryscapus nigroviolaceus* (Nees, 1834) (Hymenoptera Eulophidae) and by an unidentified *Pteromalus* (Hymenoptera Pteromalidae) (Marchesini and Dalla Montà 1994). Villemant et al. (2011) assert that in some viticultural areas of France, *T. praerogator* mainly develops at the expense of *S. pilleriana*, while in other areas it may develop even at the expense of EGVM, *E. ambiguella* and *Argyrotaenia ljungiana* (Thunberg, 1797) (= *pulchellana* Haworth, 1811).

**Table 8.** *Tranosemella praerogator* (Linnaeus): percentages of parasitism on the European grapevine moth reported in Italy by different authors.

Author/s and publication year	Italian Region/ Locality	Year	1^st^ generation (antophagous)	2^nd^ generation (carpophagous)	3^rd^ generation (carpophagous)
Marchesini and Dalla Montà 1994	Veneto/ Pernumia (PD)	1988	–	–	0
Marchesini and Dalla Montà 1994	Veneto/ Pernumia (PD)	1989	8.7	0.35	0
Marchesini and Dalla Montà 1994	Veneto/ Pernumia (PD)	1990	9.8	4.13	0
Marchesini and Dalla Montà 1994	Veneto/ Pernumia (PD)	1991	23.76	5.37	0
Marchesini and Dalla Montà 1994	Veneto/ Pernumia (PD)	1992	5.23	0.19	0
Marchesini and Dalla Montà 1994	Veneto/ Colognola (VR)	1989	–	–	0
Marchesini and Dalla Montà 1994	Veneto/ Colognola (VR)	1990	30.4	20.9	0
Marchesini and Dalla Montà 1994	Veneto/ Colognola (VR)	1991	15.56	2.08	0
Marchesini and Dalla Montà 1994	Veneto/ Colognola (VR)	1992	17.3	4.83	0
Marchesini and Dalla Montà 1994; Marchesini 2007	Veneto/ Valpolicella (VR)	1992 (1)	17.3/6.52	4.83/0	0/0
Marchesini 2006 and 2007	Veneto	2000 (2)	–	1.3/0.6	0/0
Colombera et al. 2001	Piedmont/ Caravino IPM	1998	0	0	does not occur
Colombera et al. 2001	Piedmont/ Caravino IPM	1999	0.87*	0	does not occur
Colombera et al. 2001	Piedmont/Settimo Vittone	1998	0	0	does not occur
Colombera et al. 2001	Piedmont/Settimo Vittone	1999	0.31*	0	does not occur

* recalculations on the basis of data provided by the authors.

######## Taxonomic notes.

As already mentioned by some authors (Roman 1932, Yu and Horstmann 1997, Horstmann 2006), the name “*praerogator*” Linnaeus has been used in the past to indicate *Dyspetes* Förster 1869 [*Dyspetes praerogator* Thomson, 1883 unavailable name for *D. luteomarginatus* Habermehl, 1925] (Ichneumonidae Tryphoninae). Gravenhorst (1829) attributed the Linnean species to the genus *Tryphon*, perhaps following the indication of Fabricius (1804), who assigned it to the genus *Bassus*. Roman (1932), studying the types of Linnaeus, assigned the species to the genus *Angitia* Holmgren, 1859, hypothesizing the synonymy with *Angitia armillata* (Gravenhorst) [recte *Diadegma armillata* (Gravenhorst, 1829)]. Horstmann (1973) initially assigned this species to the genus *Diadegma*, while Townes (1971) included it in the related genus *Tranosema* Förster, 1869. Later, Horstmann (1978) moved the species to the genus *Tranosemella* Horstmann.

**Figure 8.**
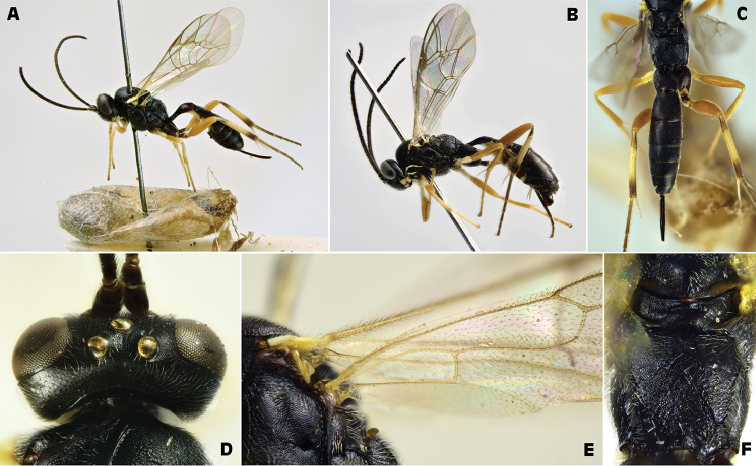
*Tranosemella praerogator* (Linnaeus, 1758) (Female: Hallig Oland, August 13–September 9, 1964, ex *Clepsis spectrana* Treitschke; male: D, Ostfriesischen Inseln, Mollum Memmert, July 27–August 3, 1985, leg. V. Haeseler; ZSM). **A** female habitus lateral view **B** male habitus lateral view **C** female propodeum and metasoma dorsal view **D** male head dorsal view **E** cells and veins of half proximal part of hind wing of female **F** female propodeum dorsal view.

####### Venturia canescens

Taxon classificationAnimaliaHymenopteraIchneumonidae

(Gravenhorst, 1829)

Venturia canescens : Marchesini and Dalla Montà 1994: 205, 1998: 3.

######## Italian distribution of reared parasitoids.

Veneto: Marchesini and Dalla Montà 1994, 1998.

######## Distribution.

The genus *Venturia* Schrottky, 1902 is represented by 136 species (Yu et al. 2012), five of which are present in Europe (Zwakhals and van Achterberg 2017). *Venturia canescens* is considered a cosmopolitan species, its distribution being related to grain trade and other stored products. In temperate and tropic areas around the world, it is most often found in buildings where grains or flour are stored (Carlson 1979).

######## Host range.

*Venturia canescens* is a koinobiont endoparasitoid that lives on larvae of various Lepidopteran species feeding on stored goods, such as *Ephestia kuehniella* (Zeller, 1879), *Plodia interpunctella* (Hübner, 1813), *Cadra* spp. Walker, *Apomyelois ceratoniae* (Zeller, 1839), *Galleria mellonella* (Linnaeus, 1758) and *Ostrinia nubilalis* (Hübner, 1796) (Pyralidae), *Nemapogon granella* (Linnaeus, 1758) (Tineidae), *Phthorimaea operculella* (Zeller, 1873) (Gelechiidae), *Prays citri* Millière, 1873 (Yponomeutidae), *Grapholita funebrana* (Treitschke, 1835) (Tortricidae) and some Noctuidae, for a total of 22 host species (Yu et al. 2012).

######## Ecological role.

*Venturia canescens* was first found associated to *L. botrana* in Veneto by Marchesini and Dalla Montà (1994), who obtained few specimens from the third generation larvae. Thiéry et al. (2001) recorded this species in the Bordeaux region, where females attack the mature caterpillar of EGVM and the larva weaves its pupal cocoon inside or outside of the host’s larval skin (Villemant et al. 2011). A *Venturia* sp. also emerged for 3^rd^ generation larvae of *L. botrana* in the Aegean Region of Turkey (Koclu et al. 2005). It is considered an occasional parasitoid of *L. botrana*, of rather marginal importance (Villemant et al. 2011). Biological, ethological, and morphological information about this species have been provided by Frilli (1965) under the name of *Devorgilla canescens*.

######## Taxonomic notes.

This species, very common and with a very wide geographical distribution, has been repeatedly described with different names and assigned to different genera. The list of synonymies and generic combinations is very long and can be found in Frilli (1965), Carlson (1979), Yu and Horstmann (1997), and Yu et al. (2012).

#### Conclusions

In this paper the records of ichneumonid parasitoids of EGVM were analyzed, belonging to the subfamilies Anomaloninae and Campopleginae. This is the first contribution on the ichneumonids associated with this pest in Italy.

Unfortunately, relatively little is known on the biology of most parasitoid species and, frequently, compilations of host-parasitoid records in literature are full of misinformations or taxonomic errors (Shaw et al. 2009). The lack of rearing protocols and/or the low accuracy in selection and managing the rearing substrates, often led to erroneous association of a parasitoid with a given host (Shaw 2017). Moreover, the endless changes occurring in taxonomy often require a critical interpretation of the names found in the literature.

Amongst the 14 taxa of ichneumonids cited in this paper, *Campoplex capitator* seems to be the best candidate to use in biological control programs against EGVM. Unfortunately, the knowledge on its behaviour and development is still not sufficient for efficient mass rearing of *C. capitator* in a bio-factory (Bagnoli and Lucchi 2006), though a recent cooperation between Italian and Chilean entomologists is promising (Lucchi et al. 2017).

So far, the host range of *C. capitator* is limited to few tortricids feeding on grapevine (Villemant et al. 2011, Yu et al. 2012), with the only exception represented by *Ancylis mitterbacheriana* (Aubert 1983). The life cicle of *C. capitator* is strongly synchronized with EGVM, with 2 to 4 generations per year moving southwards in Italy, and displaying often high parasitization rates (Tables 5 and 6).

The unsolved taxonomic confusion for the species of the genus *Campoplex* may still prevent their use in biocontrol programs and may represent an obstacle for those who are not confident with taxonomic interpretations and changes occurred in the group.

For this reason, we started to carry out a critical analysis of existing literature, conducting a direct check of voucher specimens preserved in historical collections with the aim to draw attention to possible taxonomic errors and false parasitoid-host relationships.

## Supplementary Material

XML Treatment for Agrypon flaveolatum

XML Treatment for Parania geniculata

XML Treatment for Trichomma enecator

XML Treatment for Campoplex

XML Treatment for Campoplex borealis

XML Treatment for Campoplex capitator

XML Treatment for Campoplex difformis

XML Treatment for Diadegma armillata

XML Treatment for Diadegma tenuipes

XML Treatment for Nemeritis

XML Treatment for Sinophorus crassifemur

XML Treatment for Sinophorus turionum

XML Treatment for Tranosemella praerogator

XML Treatment for Venturia canescens
